# Artificial Intelligence in Thoracic Surgery: A Review Bridging Innovation and Clinical Practice for the Next Generation of Surgical Care

**DOI:** 10.3390/jcm14082729

**Published:** 2025-04-16

**Authors:** Vasileios Leivaditis, Andreas Antonios Maniatopoulos, Henning Lausberg, Francesk Mulita, Athanasios Papatriantafyllou, Elias Liolis, Eleftherios Beltsios, Antonis Adamou, Nikolaos Kontodimopoulos, Manfred Dahm

**Affiliations:** 1 Department of Cardiothoracic and Vascular Surgery, Westpfalz Klinikum, 67655 Kaiserslautern, Germany; vnleivaditis@gmail.com (V.L.); hlausberg@westpfalz-klinikum.de (H.L.); thanospap9@yahoo.gr (A.P.); mdahm@westpfalz-klinikum.de (M.D.); 2 Department of Electrical and Computer Engineering, Democritus University of Thrace, 67100 Xanthi, Greece; amaniatopoulos@gmail.com; 3 Department of General Surgery, General Hospital of Eastern Achaia—Unit of Aigio, 25100 Aigio, Greece; 4 Department of Oncology, General University Hospital of Patras, 26504 Patras, Greece; lioliselias@yahoo.gr; 5 Department of Anesthesiology and Intensive Care, Hannover Medical School, 30625 Hannover, Germany; beltsioseleftherios@gmail.com; 6 Institute of Diagnostic and Interventional Neuroradiology, Hannover Medical School, 30625 Hannover, Germany; antonadamou@gmail.com; 7 Department of Economics and Sustainable Development, Harokopio University, 17676 Athens, Greece; nkontodi@hua.gr

**Keywords:** artificial intelligence, thoracic surgery, machine learning, robotic-assisted surgery, intraoperative guidance, postoperative care, surgical innovation, radiomics, digital twin, explainable AI (XAI)

## Abstract

**Background:** Artificial intelligence (AI) is rapidly transforming thoracic surgery by enhancing diagnostic accuracy, surgical precision, intraoperative guidance, and postoperative management. AI-driven technologies, including machine learning (ML), deep learning, computer vision, and robotic-assisted surgery, have the potential to optimize clinical workflows and improve patient outcomes. However, challenges such as data integration, ethical concerns, and regulatory barriers must be addressed to ensure AI’s safe and effective implementation. This review aims to analyze the current applications, benefits, limitations, and future directions of AI in thoracic surgery. **Methods:** This review was conducted following the Preferred Reporting Items for Systematic Reviews and Meta-Analyses (PRISMA) guidelines. A comprehensive literature search was performed using PubMed, Scopus, Web of Science, and Cochrane Library for studies published up to January 2025. Relevant articles were selected based on predefined inclusion and exclusion criteria, focusing on AI applications in thoracic surgery, including diagnostics, robotic-assisted surgery, intraoperative guidance, and postoperative care. A risk of bias assessment was conducted using the Cochrane Risk of Bias Tool and ROBINS-I for non-randomized studies. **Results:** Out of 279 identified studies, 36 met the inclusion criteria for qualitative synthesis, highlighting AI’s growing role in diagnostic accuracy, surgical precision, intraoperative guidance, and postoperative care in thoracic surgery. AI-driven imaging analysis and radiomics have improved pulmonary nodule detection, lung cancer classification, and lymph node metastasis prediction, while robotic-assisted thoracic surgery (RATS) has enhanced surgical accuracy, reduced operative times, and improved recovery rates. Intraoperatively, AI-powered image-guided navigation, augmented reality (AR), and real-time decision-support systems have optimized surgical planning and safety. Postoperatively, AI-driven predictive models and wearable monitoring devices have enabled early complication detection and improved patient follow-up. However, challenges remain, including algorithmic biases, a lack of multicenter validation, high implementation costs, and ethical concerns regarding data security and clinical accountability. Despite these limitations, AI has shown significant potential to enhance surgical outcomes, requiring further research and standardized validation for widespread adoption. **Conclusions:** AI is poised to revolutionize thoracic surgery by enhancing decision-making, improving patient outcomes, and optimizing surgical workflows. However, widespread adoption requires addressing key limitations through multicenter validation studies, standardized AI frameworks, and ethical AI governance. Future research should focus on digital twin technology, federated learning, and explainable AI (XAI) to improve AI interpretability, reliability, and accessibility. With continued advancements and responsible integration, AI will play a pivotal role in shaping the next generation of precision thoracic surgery.

## 1. Introduction

Artificial intelligence (AI) is revolutionizing medicine by facilitating the analysis of complex data, supporting clinical decision-making, and enhancing the accuracy of diagnostic and therapeutic procedures. Through machine learning algorithms, natural language processing, and computer vision, AI enables early disease detection, accelerates treatment processes, and paves the way for personalized healthcare, thereby significantly improving patient outcomes [1]. This review seeks to answer the research question of how current artificial intelligence technologies are being applied across the thoracic surgical pathway, and what their benefits, limitations, and future implications for clinical integration are.

Thoracic surgery—encompassing the management of disorders affecting the lungs, esophagus, mediastinal structures, and chest wall—presents unique challenges due to the complexity of anatomical structures and the high precision required during interventions. In this setting, AI has emerged as a promising tool for refining surgical techniques, enhancing diagnostic accuracy, and improving postoperative care [2,3,4]. Consequently, this review provides a comprehensive analysis of current AI applications in thoracic surgery.

Over the past century, surgical practices have evolved from traditional open procedures to minimally invasive techniques. Innovations such as video-assisted thoracic surgery (VATS) and robotic-assisted thoracic surgery (RATS) have replaced many conventional open thoracotomies, leading to reduced incision sizes, diminished postoperative pain, shorter hospital stays, and quicker recovery times [5,6]. Nonetheless, the limited surgical exposure inherent in these methods can compromise visibility and instrument maneuverability, thereby necessitating advanced imaging and precision-guided tools [7].

To address these challenges, the integration of AI into surgical practice has become increasingly vital. Recent advancements have demonstrated that AI-driven computer models and machine learning methods can process extensive imaging datasets to accurately identify anatomical structures and detect abnormalities, thereby supporting preoperative planning and intraoperative decision-making [8,9]. The field of AI encompasses several modalities with distinct but complementary applications in thoracic surgery, including machine learning (ML), deep learning (DL), computer vision, natural language processing (NLP), and reinforcement learning. These technologies support a wide range of functions—from diagnostic imaging and pattern recognition to surgical planning, intraoperative navigation, and postoperative monitoring. The rapidly expanding body of literature attests to the growing interest in this field. For instance, a recent narrative review examined the integration of machine learning in lung surgery, demonstrating its potential in diagnosis, preoperative risk assessment, surgical planning, and outcome prediction. Despite these promising applications, the low implementation rate in clinical practice—largely due to the technology’s novelty and associated challenges—highlights a significant gap in the literature and stresses the need for further research [10]. The evolution of AI in surgical practice is particularly relevant to the limitations faced by VATS and RATS, which, despite offering minimally invasive advantages, are constrained by restricted visualization, limited tactile feedback, and complex learning curves. AI has emerged as a tool for overcoming these barriers by enhancing precision, augmenting intraoperative awareness, and optimizing surgical workflows.

Given the critical importance of precision and safety in minimally invasive thoracic surgery, AI offers a valuable means of overcoming traditional limitations by providing real-time analysis and actionable insights [6,11]. This review aims to critically evaluate the current literature on AI applications in thoracic surgery, covering its role throughout the entire surgical pathway—from early diagnosis to postoperative care. Specifically, it explores AI-driven advancements in diagnostic imaging and risk stratification, highlighting its integration into lung cancer screening, nodule detection, and staging through radiomics, deep learning models, and biomarker analysis. Additionally, this review examines AI’s contribution to surgical decision-making and intraoperative precision, particularly in RATS and VATS techniques, where AI-powered navigation, augmented reality, and real-time tissue recognition systems enhance surgical accuracy and safety.

Beyond the intraoperative phase, AI applications in postoperative care and patient monitoring are evaluated, including predictive analytics for complication risk, AI-assisted rehabilitation protocols, and machine-learning-based models for prognosis assessment. Furthermore, this review assesses AI’s emerging role in oncological therapies, such as chemotherapy, immunotherapy, and radiotherapy, where it aids in treatment response prediction, precision medicine, and toxicity minimization. By synthesizing these multidisciplinary advancements, this study not only consolidates current knowledge but also identifies existing challenges—such as data standardization, model interpretability, clinical integration, and ethical considerations—that hinder broader AI implementation in thoracic surgery. Ultimately, this review seeks to provide valuable insights for clinicians, researchers, and policymakers, guiding future innovation and encouraging the responsible adoption of AI in thoracic surgical practice.

## 2. Materials and Methods

### 2.1. Critical Analysis of Bias

The reliability and applicability of the findings in this review are significantly influenced by various forms of bias. Many of the included studies relied on non-randomized or retrospective datasets, leading to selection bias, as certain patient populations or disease stages were overrepresented, thereby limiting the generalizability of the findings [12]. Furthermore, reporting bias was frequently encountered, with some studies lacking comprehensive descriptions of their AI methodologies, particularly regarding model development, training, and validation processes. This insufficient transparency hinders reproducibility, makes critical evaluations more difficult, and affects the overall credibility of the reported results [13].

Publication bias was also identified, as studies demonstrating positive outcomes were disproportionately represented, whereas those reporting negative or inconclusive findings were underreported. Such selective reporting may overestimate the effectiveness of AI applications in thoracic surgery. Additionally, performance and detection biases were prevalent due to the wide variation in training and validation datasets, with many studies relying on small, institution-specific datasets. This raises concerns about overfitting, which reduces the external validity and clinical applicability of AI models in broader healthcare settings [14].

These biases collectively impact the clinical implementation and scalability of AI technologies in thoracic surgery. Selection and performance biases reduce external validity, making it challenging to generalize findings across diverse patient populations. Similarly, inadequate methodological transparency resulting from reporting bias impairs the ability to systematically assess the reliability of AI-driven solutions in real-world surgical settings. A structured overview of the main biases identified across the included studies, along with their implications and mitigation strategies, is provided in Table 1.

To systematically assess and mitigate these biases, validated instruments such as the Cochrane Risk of Bias Tool (for randomized controlled trials), ROBINS-I (for non-randomized studies), and QUADAS-2 (for diagnostic accuracy studies) were employed [15]. Based on these tools, the majority of the included studies were classified as having a moderate risk of bias, mainly due to limitations in dataset quality, methodological transparency, and reporting standards. Addressing these shortcomings in future research will be critical for improving the reliability and clinical translation of AI applications in thoracic surgery.

While the risk of bias was assessed and classified using established tools (Cochrane Risk of Bias Tool, ROBINS-I, and QUADAS-2), we also addressed bias considerations in the synthesis and discussion of results. Studies with a higher risk of bias were interpreted with caution, and their findings are contextualized accordingly in the Discussion section.

Heterogeneity among studies—stemming from differences in AI models, surgical applications, datasets, and outcome metrics—precluded formal meta-analysis. Instead, a qualitative synthesis approach was used, allowing for thematic grouping and a descriptive comparison of results across domains (e.g., diagnostics, intraoperative support, postoperative care). This approach allowed us to identify trends, strengths, and limitations without overgeneralizing findings across heterogeneous methodologies.

### 2.2. Inclusion and Exclusion Criteria

This review was conducted in accordance with the Preferred Reporting Items for Systematic Reviews and Meta-Analyses (PRISMA) guidelines to ensure transparency, reproducibility, and methodological rigor. The primary objective was to comprehensively evaluate the role of AI in thoracic surgery, with a focus on its applications in surgical techniques, postoperative care, oncological treatments, and early diagnosis [16].

To identify relevant literature published up to January 2025, a comprehensive search was conducted across multiple electronic databases, including PubMed, Scopus, Web of Science, and the Cochrane Library. The search strategy incorporated Medical Subject Headings (MeSH) terms and keywords, such as thoracic surgery, artificial intelligence, minimally invasive procedures, oncology, and machine learning, which were combined using Boolean operators (AND, OR) to optimize sensitivity and specificity, and the following core terms and their combinations were applied: “artificial intelligence” OR “machine learning” OR “deep learning” OR “computer vision” OR “natural language processing” AND “thoracic surgery” OR “minimally invasive thoracic surgery” OR “VATS” OR “RATS” OR “lung cancer surgery”.

Only articles published in English were considered for inclusion due to resource constraints related to translation. No geographic or institutional limitations were applied. The search did not include grey literature (e.g., conference abstracts, theses, or non-peer-reviewed reports), as the focus was on peer-reviewed, methodologically sound primary studies and systematic reviews.

#### 2.2.1. Inclusion Criteria

Studies were included if they met the following criteria:

- Peer-reviewed publications on the application of AI in thoracic surgery.
- English-language articles.
- Research examining AI applications in thoracic surgery for diagnostic, surgical, therapeutic, or postoperative care purposes.
- Systematic reviews, meta-analyses, or original research presenting primary data.
- Studies with clearly defined methodologies, AI models, and measurable outcomes relevant to thoracic surgery.

#### 2.2.2. Exclusion Criteria

The following types of studies were excluded:

- Articles focusing exclusively on preclinical or animal models.
- Studies unrelated to minimally invasive techniques or thoracic surgery.
- Abstracts, conference proceedings, editorials, opinion pieces, or gray literature.
- Research that did not explicitly apply AI or lacked sufficient methodological detail.

To minimize errors and enhance consistency, two independent reviewers (blinded to each other’s assessments) screened all identified studies. Data extraction was performed systematically, collecting information on the following:

- Study design.
- AI methodology and model architecture.
- Application focus (e.g., diagnosis, surgery, postoperative care, treatment).
- Sample size and patient demographics.
- Outcome measures and performance metrics.
- Study limitations and potential biases.

To assess methodological quality and the risk of bias, validated tools were employed, including ROBINS-I for non-randomized studies and the Cochrane Risk of Bias Tool for randomized controlled trials. Each study was classified as having a low, moderate, or high risk of bias based on criteria such as selection bias, outcome measurement, and completeness of reporting [17].

This review was conducted in accordance with the Preferred Reporting Items for Systematic Reviews and Meta-Analyses (PRISMA) guidelines. No prior registration was undertaken, as the objective of this work was not to systematically accumulate or quantitatively synthesize clinical or patient-level data. Instead, the review provides a conceptual and methodological synthesis of current and emerging applications of artificial intelligence in thoracic surgery. Given its theoretical scope, focusing on innovation pathways, clinical integration challenges, and future perspectives rather than patient interventions or treatment outcomes, formal registration in a database such as PROSPERO was deemed unnecessary and not applicable.

### 2.3. Study Selection Process

**Figure 1:**
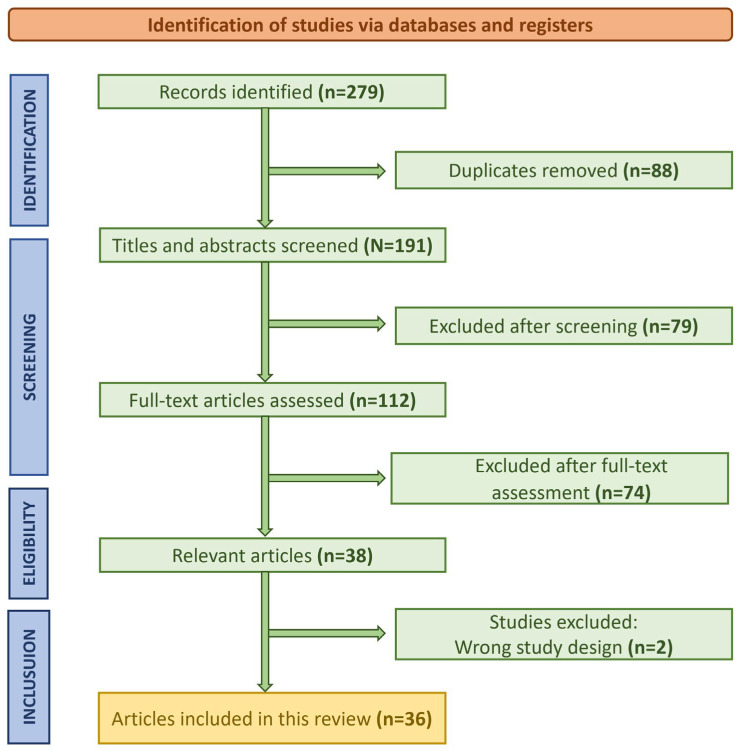
PRISMA (Preferred Reporting Items for Systematic Reviews and Meta-Analyses) flowchart illustrating the study selection process for this review. The chart details the number of studies identified through database searches, screened for relevance, and assessed for eligibility, leading to the final inclusion of 36 studies in the qualitative synthesis.

A total of 279 studies were identified through the initial database search. After the removal of duplicates and screening of titles and abstracts, 112 studies were deemed eligible for full-text review. Following the application of inclusion and exclusion criteria, a final set of 36 studies was included in the qualitative synthesis. The PRISMA flowchart (Figure 1) outlines the study selection process. The final set of 36 included studies is summarized in Table 2, including key information and main findings.

### 2.4. Discussion and Gaps in the Literature

**Figure 2:**
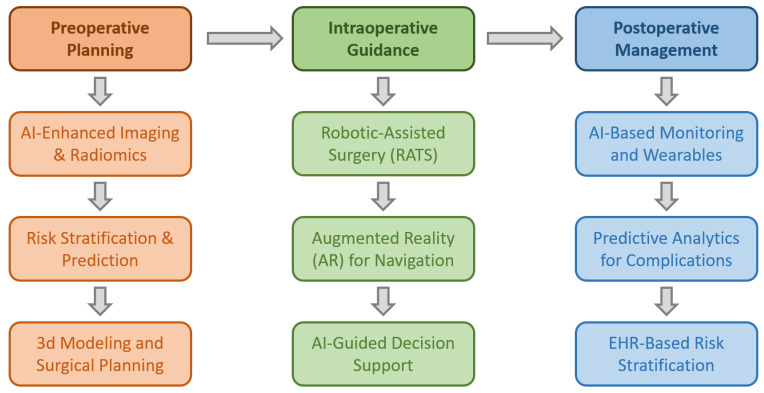
The role of artificial intelligence (AI) across different phases of thoracic surgery. In the preoperative phase, AI enhances imaging through radiomics, assists in risk stratification, and supports 3D modeling for surgical planning. During the intraoperative phase, AI-driven robotic-assisted surgery, augmented reality (AR) navigation, and AI-guided decision support improve surgical precision and efficiency. In the postoperative phase, AI-based monitoring systems, predictive analytics for complications, and electronic health record (EHR)-based risk stratification contribute to enhanced recovery and long-term patient management. This figure illustrates the integration of AI throughout the surgical pathway to optimize patient outcomes and clinical decision-making.

The collective findings from this review highlight the transformative potential of AI in thoracic surgery, particularly in early diagnosis, surgical precision, and postoperative care. AI models have demonstrated increased diagnostic accuracy in detecting lung lesions, identifying lymph node metastases, and enhancing risk stratification. Additionally, robotic platforms incorporating AI have improved surgical precision, minimized human error, and reduced the risk of complications. However, significant heterogeneity in study design, AI methodologies, and datasets poses challenges in directly comparing findings across studies and establishing uniform performance benchmarks for AI applications in thoracic surgery [30]. The main findings of this review are briefly presented in Figure 2.

Despite these promising advancements, several critical gaps persist in the literature. One of the most significant challenges is the limited generalizability of AI models due to the reliance on small, institution-specific datasets, which often lack diversity in patient demographics and disease presentations. A key barrier to wider clinical adoption remains the absence of large-scale, multicentric, and well-annotated datasets, which are essential for developing robust, externally validated AI models [50].

Moreover, issues surrounding AI model interpretability and transparency continue to hinder trust and adoption in clinical settings. The lack of standardized reporting guidelines for AI methodologies, training datasets, and performance metrics further complicates study comparability and reproducibility. In addition, most existing studies are retrospective, with few prospective validations of AI applications. Research gaps also extend to the long-term clinical outcomes of AI-driven interventions, as well as ethical and regulatory concerns, including patient data protection, AI bias, and approval processes for AI-based technologies in thoracic surgery [51,52].

To address these challenges, future research should prioritize the development of large, diverse, and annotated datasets through international collaborative efforts. Implementing explainable AI (XAI) techniques will be crucial for enhancing model transparency and fostering trust among clinicians. Additionally, the adoption of standardized reporting frameworks, such as TRIPOD-AI (for risk prediction models) and CONSORT-AI (for clinical trials involving AI), will improve the consistency and quality of AI research. Furthermore, multicenter trials are essential to validate AI applications across different patient populations and healthcare systems.

Lastly, the exploration of advanced AI methodologies, such as digital twins (patient-specific simulations for surgical planning) and federated learning (privacy-preserving AI training across multiple institutions), may help overcome current limitations and expand AI applications in thoracic surgery. These advancements will reinforce the evidence base for AI integration in thoracic surgery, paving the way for safer, more effective, and ethically responsible implementation in clinical practice [53].

## 3. Results

### 3.1. Early Diagnosis of Thoracic Pathologies

**Figure 3:**
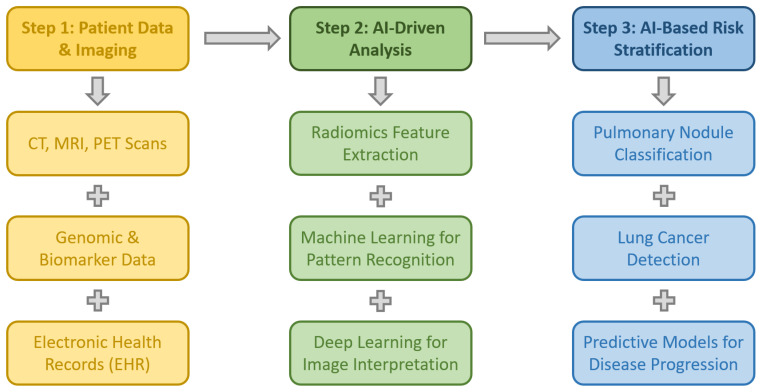
The role of AI in the early diagnosis of thoracic pathologies, particularly lung cancer. The process begins with patient data collection and imaging, including CT, MRI, PET scans, genomic biomarkers, and electronic health records (EHRs). AI-driven analysis is then applied to extract radiomics features, recognize patterns through machine learning, and interpret imaging using deep learning models. Finally, AI-based risk stratification assists in pulmonary nodule classification, lung cancer detection, and disease progression prediction, supporting early intervention and personalized treatment planning. This framework enhances diagnostic accuracy, facilitates early detection, and improves clinical decision-making in thoracic oncology.

Early diagnosis is crucial in thoracic diseases, particularly for lung cancer, which remains a leading cause of cancer-related mortality worldwide. AI-driven technologies have significantly improved the accuracy and efficiency of early detection by integrating genomic data, biomarkers, and radiological imaging. These advanced AI algorithms enhance diagnostic precision, enabling earlier interventions, personalized treatment strategies, and improved patient outcomes [10,54]. Figure 3 diagrammatically presents the role of AI in the early diagnosis of thoracic pathologies.

One notable advancement is LungDiag, an AI-based diagnostic system developed by Liang et al., which employs natural language processing (NLP) to extract critical clinical data from electronic health records (EHRs) [22]. In a multicenter study, the model demonstrated exceptional diagnostic accuracy, achieving an F1 score of 0.711 for the top diagnosis and 0.927 for the top three diagnoses. The study trained the AI model using 31,267 EHRs and validated its performance with 1142 EHRs from three external centers, confirming its robust real-world applicability. Notably, LungDiag outperformed both ChatGPT 4.0 and human specialists in diagnostic accuracy, underscoring its potential to assist physicians in more precise and efficient diagnosis of respiratory diseases. Despite these promising results, further large-scale validation is necessary to assess LungDiag’s generalizability across diverse patient demographics and healthcare settings. Ensuring broad clinical applicability will require prospective studies to evaluate its real-world effectiveness and its integration into routine clinical workflows.

#### 3.1.1. Screening and Early Detection and Evaluation of Pulmonary Nodules

The early detection of pulmonary nodules is critical for improving lung cancer prognosis, as timely identification and classification allow for earlier intervention and tailored treatment strategies. AI has demonstrated considerable potential in this domain by enhancing nodule detection, segmentation, and characterization through advanced machine learning algorithms, particularly deep learning and convolutional neural networks (CNNs). AI-based clinical decision-support systems (CDSSs) are increasingly being integrated into diagnostic workflows to improve risk stratification and treatment planning, although challenges such as model interpretability, data standardization, and clinical validation remain key concerns [19,54].

##### AI in Lung Cancer Screening and Nodule Detection

Zhang and Chen provide an extensive review of AI applications in lung cancer screening, diagnosis, and treatment, highlighting the role of computer vision for image analysis and feature engineering for structured data processing. The authors emphasize AI’s contributions to genetic and imaging-based diagnosis, pathological analysis, automated nodule detection, and identification of high-risk populations. They further discuss the importance of explainable machine learning, transfer learning, and federated learning in addressing the challenges associated with AI adoption, such as the lack of annotated datasets and the restricted interpretability of deep learning models. Their study underlines AI’s transformative potential in lung cancer care while calling for further research on model transparency and data privacy to facilitate broader clinical adoption [19].

Chassagnon et al. examine the evolution of AI-driven computer-aided detection (CADe) systems, which have been commercially available since the early 2000s, and their role in pulmonary nodule detection. The authors discuss how deep learning has revolutionized thoracic imaging and how AI is now being actively integrated into clinical workflows. Their review highlights AI’s ability to enhance diagnostic accuracy, support personalized treatment plans, and ultimately improve patient outcomes, marking a transition from theoretical AI applications to practical implementation in clinical practice [54].

##### AI-Assisted Detection and Follow-Up of Indeterminate Pulmonary Nodules (IPNs)

Woodhouse et al. investigated the application of natural language processing (NLP)-driven AI systems in identifying clinically significant, indeterminate pulmonary nodules (IPNs) that are incidentally discovered on computed tomography (CT) scans. The study analyzed over 76,000 radiology reports from a single tertiary care center and flagged 389 IPNs, with 30% of cases lacking appropriate follow-up per guideline recommendations. By identifying these nodules, the AI-assisted system ensured that additional clinical consultations and three surgical interventions were scheduled, thereby improving patient management and adherence to standard care guidelines. These findings highlight AI’s potential role as a safety net in clinical practice, helping prevent missed or delayed diagnoses of malignant nodules [21].

Similarly, Zolfaghari, Kuehne, and Antonoff discussed the integration of AI in incidental lung nodule screening, emphasizing its ability to detect nodules overlooked during routine imaging. They argue that AI-based systems enhance workflow efficiency by flagging potentially clinically significant findings, ensuring that they receive timely medical evaluation and follow-up. Their study reinforces AI’s emerging role as an essential component of early lung cancer detection strategies and a means of reducing diagnostic oversight [55].

##### Advancements in AI for Lung Nodule Characterization

Cellina et al. explore the transformative role of AI in lung cancer imaging, particularly in lesion detection and segmentation. Their study highlights how AI-driven image processing can reduce interobserver variability, accelerate diagnostic workflows, and improve overall diagnostic accuracy. The review mentions the importance of AI in enhancing precision in lung cancer detection and treatment planning, which is critical for minimizing unnecessary invasive procedures and improving risk stratification [56].

Yang et al. provide a detailed analysis of deep learning applications in pulmonary nodule diagnosis, focusing on CNN-based models for feature extraction, nodule classification, and false-positive reduction. Their findings suggest that deep learning can significantly improve diagnostic accuracy and efficiency, although challenges related to model interpretability and the need for large, high-quality annotated datasets remain barriers to widespread adoption [57].

##### AI in Lung Cancer Screening Programs and Risk Stratification

Adams et al. assess the role of AI in lung cancer screening programs, referencing findings from the National Lung Screening Trial (NLST) and the NELSON trial, which demonstrated that low-dose CT screening reduces lung cancer mortality. Their review discusses the importance of risk stratification, highlighting how AI can help identify high-risk individuals, optimize screening intervals, and integrate smoking cessation programs into lung cancer prevention strategies. They also evaluate the cost-effectiveness of AI-driven screening models, proposing potential solutions for global implementation [58].

In a separate study, Adams et al. examine the role of ML in lung cancer screening, emphasizing its ability to reduce radiation doses, improve image quality, and optimize detection rates. Their research highlights AI’s capacity to automate risk assessments and integrate findings from various chronic disease screenings, potentially offering opportunistic screening solutions to enhance population health and promote health equity in lung cancer care [59].

##### AI in Lung Nodule Diagnosis and Staging

De Margerie-Mellon and Chassagnon evaluate the role of deep learning and CNNs in automating lung nodule detection and classification on chest X-rays and CT scans. Their findings suggest that AI models now perform at or above human radiologist levels in detecting malignant versus benign nodules. They also highlight AI’s role in non-invasive tumor characterization, including histological subtyping, genetic mutation prediction, and prognosis assessment. However, challenges related to AI generalizability, a lack of interpretability, and insufficient clinical validation remain obstacles to full-scale implementation in routine clinical practice [60].

Wu et al. further explore AI’s role in lung cancer screening, particularly in radiomics-based segmentation, false-positive reduction, and malignancy classification. Their review emphasizes AI’s ability to predict tumor growth and identify genetic and pathological subtypes, which can refine individualized treatment planning. However, the authors caution that standardization, large-scale validation, and improved model interpretability are necessary for AI to reach its full clinical potential [61]. These conclusions are supported by the findings of Quannyang et al. who support the use of AI in lung cancer screening, enhancing nodule detection sensitivity, reducing false-positive rates, and classifying nodules [62].

#### 3.1.2. Radiomics and AI in Lung Cancer Diagnosis

Radiomics is an advanced imaging analysis technique that extracts quantitative features from medical images such as CT, PET, and MRI to enhance lung cancer diagnosis and treatment [63,64]. By analyzing texture, shape, intensity, and spatial relationships within lung lesions, radiomics enables more precise tumor characterization beyond conventional imaging. It aids in differentiating benign from malignant nodules, identifying histological subtypes, and predicting genetic mutations (e.g., EGFR, ALK) essential for targeted therapies. Additionally, radiomics assesses tumor heterogeneity, providing prognostic insights and guiding personalized treatment. When integrated with artificial intelligence (AI) and machine learning, radiomics supports non-invasive, data-driven decision-making, improving early detection, risk stratification, and individualized therapy planning [65,66].

Chen et al. reviewed radiomics–AI integration in precision medicine, emphasizing its ability to capture tumor characteristics beyond human perception. AI-enhanced radiomic models predict histological and molecular profiles, stratify patient risk, and forecast clinical outcomes, offering a cost-effective, reproducible alternative to tissue biopsies. The study highlights personalized therapy applications while identifying challenges such as data standardization and clinical validation [63]. Liu et al. developed a radiomics-based nomogram to distinguish benign from malignant pulmonary nodules non-invasively [67]. Their retrospective study utilized radiomic features from CT scans, selecting 20 key variables through the LASSO method for a logistic regression model. The nomogram showed high diagnostic accuracy, supporting its potential role in early lung cancer detection and treatment planning.

#### 3.1.3. Bronchoscopy, Ultrasound, and Endobronchial Ultrasound (EBUS)

AI is increasingly integrated into bronchoscopy and ultrasound-based diagnostics, enhancing procedural accuracy and real-time decision-making while addressing limitations in traditional approaches. AI-powered systems are improving lung cancer detection, lymph node assessment, and operator performance in bronchoscopy, lung ultrasound, and EBUS, though challenges related to interpretability, validation, and clinical adoption remain [68].

##### AI in Bronchoscopy and Lung Cancer Diagnosis

Mehta explores AI’s role in bronchoscopy, emphasizing its ability to improve diagnostic yield, procedural precision, and operator assistance. However, he warns against over-reliance on AI, citing concerns over data privacy, operator dependence, and algorithm validation. He advocates for comprehensive training programs to ensure clinicians effectively integrate AI without compromising their procedural expertise [68].

Zhang and Yu investigated AI’s application in small-cell lung cancer (SCLC) pathology using fiberoptic bronchoscopy and hesperetin derivatives. Their AI-assisted hesperetin–fiber bronchoscope study (H-FBS) achieved a 97.9% diagnostic accuracy, outperforming traditional biopsy methods (89%). This study highlights AI’s potential to streamline and enhance SCLC diagnosis while offering a cost-effective alternative to multiple circulating tumor cell (CTC) detections [69].

##### AI in Lung Ultrasound for Thoracic Surgery

Števík et al. conducted a prospective study to assess AI’s accuracy in detecting A-lines on lung ultrasound in patients undergoing thoracic surgery. Their hybrid AI model (86.6% accuracy) outperformed a radiology resident (55.8%), though the resident’s performance improved significantly with AI feedback. The AI system demonstrated superior sensitivity (92.8%) and specificity (83.4%), suggesting its potential for enhancing diagnostic accuracy and training radiology residents through real-time support [20].

##### AI in Endobronchial Ultrasound (EBUS) for Lymph Node Metastasis

Ishiwata et al. developed a deep learning model using the SqueezeNet architecture to predict lymph node metastases from EBUS images. Their model, trained on automatically extracted EBUS frames, achieved 91.1% accuracy and 96.7% sensitivity, significantly outperforming conventional stochastic gradient descent methods. The study suggests that AI-enhanced EBUS imaging can improve lung cancer staging and guide treatment decisions, though large-scale prospective validation remains necessary [25].

Churchill et al. introduced NeuralSeg, an AI model trained to segment and analyze lymph nodes in EBUS imaging. The study demonstrated AI’s potential to predict lymph node metastases, supporting its use in mediastinal staging to enhance diagnostic precision and clinical decision-making [70].

Patel et al. further evaluated NeuralSeg’s performance in 187 lymph nodes from 124 patients undergoing EBUS-guided transbronchial needle aspiration (EBUS-TBNA). The AI model, which assessed lymph node stiffness via elastography, achieved a diagnostic accuracy of 70.59%, with high specificity (90.74%) but moderate sensitivity (43.04%). While the findings suggest that AI can refine lung cancer staging, the authors recommend further refinement through multicenter trials to improve clinical utility [71].

### 3.2. AI in Lung Cancer Staging

**Figure 4:**
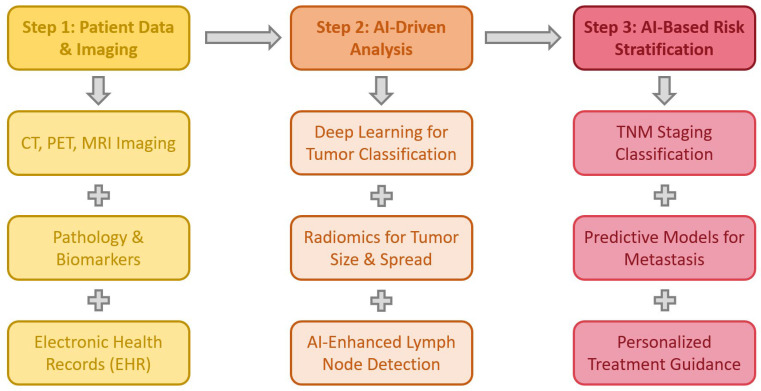
The role of AI in lung cancer staging, integrating multimodal data analysis with predictive modeling. The process begins with multimodal data collection, incorporating CT, PET, and MRI imaging, pathology and biomarker assessments, and electronic health records (EHRs). AI-driven analysis then applies deep learning for tumor classification, radiomics for tumor size and spread assessment, and AI-enhanced lymph node detection to refine staging accuracy. Finally, AI-based decision support assists in TNM staging classification, metastasis prediction, and personalized treatment guidance, improving precision in diagnosis, prognosis, and therapeutic planning for lung cancer patients.

Accurate staging of lung cancer is crucial for determining treatment strategies and prognosis. AI, particularly deep learning and radiomics models, has shown promise in improving staging accuracy by analyzing imaging, clinical data, and biomarkers. Several studies highlight AI’s role in lymph node assessment, metastatic prediction, and survival stratification [72]. Figure 4 summarizes the potential role of AI in lung cancer staging.

#### 3.2.1. AI in Imaging-Based Staging

Zheng et al. conducted a systematic review and meta-analysis evaluating the diagnostic accuracy of deep learning and radiomics models in lung cancer staging. Their analysis of 19 eligible studies (14 using radiomics, 5 employing deep learning) reported pooled AUROC values of 0.83 for lung cancer detection, 0.78 for NSCLC identification, 0.79 for malignant nodule differentiation, and 0.74 for lymph node metastasis prediction. These findings suggest that AI models can enhance staging accuracy and clinical decision-making in lung cancer care [72].

#### 3.2.2. AI-Based Prediction of Lymph Node Metastases

Yoshimura et al. developed an AI model to predict lymph node metastases in 988 NSCLC patients undergoing mediastinal lymph node dissection and lung resection. Their study applied six machine learning algorithms, with gradient boosting (GB) achieving the highest accuracy (80.0% accuracy, 95.6% specificity). This AI model provides a non-invasive alternative to PET or contrast-enhanced CT, helping guide surgical planning without additional imaging [24].

Zhong et al. developed a deep learning model to predict N2 lymph node metastasis and overall survival in stage I NSCLC patients based on preoperative CT imaging. The model effectively identified high-risk patients, aiding in surgical decision-making and personalized prognostication, highlighting AI’s potential in refining early-stage lung cancer management [73].

#### 3.2.3. AI in Multidisciplinary Lung Cancer Management

Ladbury et al. reviewed AI’s role in lung cancer diagnosis, staging, and treatment planning, emphasizing its ability to personalize therapy and predict patient outcomes by integrating imaging, pathology, and clinical data. The authors stress the importance of AI-driven multidisciplinary collaboration, as well as challenges such as data harmonization, model validation, and institutional cooperation to ensure AI’s effective clinical implementation [74].

### 3.3. Risk Assessment

Accurate risk assessment and staging are essential for predicting patient outcomes and guiding personalized treatment strategies. AI models integrating clinical, imaging, and molecular data enhance risk stratification, surgical planning, and prognostic evaluation, supporting evidence-based decision-making [75].

#### 3.3.1. AI in Preoperative Risk Assessment

Poullis explored AI’s impact on risk modeling in thoracic and cardiac surgery, emphasizing its superiority over traditional statistical methods in analyzing large datasets and predicting patient outcomes. He highlights AI’s ability to enhance preoperative risk evaluation and treatment personalization, while noting challenges such as data integration, model interpretability, and validation [75]. Wu et al. conducted a systematic review of lung cancer risk prediction models, assessing their applicability in clinical settings. They emphasize the need to integrate clinical and imaging data with advanced computational techniques to enhance malignancy predictions, while also calling for external validation and standardization to improve model generalizability [61]. Harris and Matthews explore the role of AI in preoperative risk assessment, emphasizing its ability to predict perioperative complications and enhance surgical decision-making. Their study highlights how AI-driven models analyze patient-specific factors, including comorbidities, imaging data, and laboratory results, to provide personalized risk stratification. AI’s predictive capabilities can aid in identifying high-risk patients, optimizing perioperative management, and improving surgical outcomes. However, the authors stress the importance of rigorous validation, clinician oversight, and ethical considerations to ensure safe and effective AI integration into preoperative assessments [76].

#### 3.3.2. AI Models for Postoperative Mortality and Survival Prediction

Raghu et al. developed CXR-CTSurgery, a deep learning model trained on 9283 patients to predict postoperative mortality using preoperative chest radiographs. The model’s performance was comparable to the Society of Thoracic Surgeons Predicted Risk of Mortality (STS-PROM) scores. For non-standard procedures, it achieved higher accuracy, suggesting AI’s utility in risk assessment where traditional models lack guidance [77]. Lynch et al. applied ML techniques to the SEER database, using tumor characteristics, demographics, and clinical parameters to predict survival in lung cancer patients. Their ensemble model, with gradient boosting as the most influential component, achieved an RMSE of 15.05 months, demonstrating AI’s potential in individualized survival estimation [78]. She et al. introduced a deep learning survival neural network (DSNN) trained on 12,958 NSCLC patients, outperforming the tumor–node–metastasis (TNM) staging system. The model’s superior predictive accuracy suggests its clinical utility in guiding treatment decisions [79].

#### 3.3.3. AI in Surgical Risk Stratification

Esteva et al. discuss the role of artificial neural networks (ANNs) in predicting surgical outcomes, emphasizing AI’s ability to provide personalized, data-driven risk assessments. They caution that heterogeneous retrospective datasets can impact model validity, but highlight AI’s potential as a decision-support tool in thoracic surgery [47]. Seastedt et al. conducted a scoping review on ML applications in thoracic surgery, identifying AI’s benefits in preoperative test accuracy, pathology diagnosis, and postoperative risk assessment. However, they note concerns regarding model calibration, reproducibility, and algorithmic bias, advocating for greater surgeon involvement in AI integration [46].

#### 3.3.4. AI in Personalized Prognostic Assessment

Zhong et al. developed a cross-modal deep learning model using PET/CT imaging to predict occult nodal metastasis (ONM) in stage N0 NSCLC patients. The model, trained on 1039 patients, achieved AUC values of 0.87 (training) and 0.84 (validation), demonstrating its potential as a non-invasive tool for preoperative risk assessment [80]. Lococo et al. reviewed AI’s role in personalized prognosis for NSCLC, emphasizing its ability to integrate clinical, molecular, and imaging data to predict disease progression, treatment response, and recurrence patterns. They highlight challenges in data quality, model interpretability, and regulatory approval, stressing the need for interdisciplinary collaboration to ensure AI’s responsible clinical adoption [81].

### 3.4. AI in Predicting Outcomes for Lung Transplantation

AI-driven models are increasingly being explored to enhance donor–recipient matching, predict post-transplant complications, and improve long-term survival outcomes in lung transplantation. By integrating deep learning, machine learning, and computer vision, AI has the potential to optimize lung size estimation, survival prediction, and rejection risk assessment, ultimately improving patient outcomes [27,28,29,31].

#### 3.4.1. AI for Lung Size Estimation and Donor Matching

Ismail et al. conducted a pilot study using deep learning and computer vision to develop an AI-based automated lung size estimation system from portable chest radiographs. The system extracted lung masks and key feature points to determine six different height and width measurements, which were validated against expert radiologist assessments in 50 lung transplant recipients. The AI system demonstrated strong interrater and intrarater agreement, with a measurement error of less than 2.5% (≤7.0 mm), even in challenging cases involving consolidations, effusions, or patient rotation. While further validation in larger cohorts is needed, this study highlights AI’s potential to provide standardized, reliable lung size measurements for better donor–recipient matching and broader clinical applications [27].

#### 3.4.2. AI in Predicting Post-Transplant Complications and Survival

Sargiotis et al. conducted a systematic review evaluating AI models for predicting post-transplant complications and long-term survival in heart and lung transplant recipients. Their analysis of 15 eligible studies found that AI models demonstrated strong predictive performance. Random forest and extreme gradient boosting models outperformed traditional linear approaches, though concerns were raised regarding sample homogeneity, as most studies focused on North American and White populations, excluding pediatric recipients. Additionally, a high risk of bias was observed in several studies, emphasizing the need for more diverse and externally validated datasets [28].

Tian et al. developed a random survival forest (RSF) model to predict overall survival after lung transplantation (LTx) in a retrospective study of 504 patients. The model, trained on 353 patients and tested on 151, outperformed traditional Cox regression models. The RSF model identified postoperative ECMO duration as the most significant survival predictor, effectively stratifying patients into high- and low-risk groups with a mean survival of 14.83 vs. 52.91 months. These findings suggest that AI models could enhance risk stratification and long-term prognostic assessments in lung transplant recipients [29].

#### 3.4.3. AI for Non-Invasive Rejection Monitoring

Shigemura et al. discuss the limitations of transbronchial biopsies, the gold standard for detecting acute lung allograft rejection, citing institutional variations in biopsy techniques and high procedural costs [31]. A multicenter study by Todd et al. (2026 biopsies from 400 recipients) reported an acute rejection rate of 53.3%, with transplant type and HLA mismatch as major risk factors. Given the invasiveness, cost, and risk of sampling errors, the authors advocate for AI-driven diagnostic approaches to enhance precision, reduce procedural risks, and improve alloimmune response predictions in lung transplant recipients [33].

### 3.5. AI in Genomics, Proteomics, and Novel Therapeutic Targeting

AI is transforming oncology research and drug discovery, offering powerful tools for target identification, genomic analysis, and precision medicine. By integrating machine learning, deep learning, and network-based approaches, AI enhances our ability to detect genetic mutations, classify tumors, and predict therapeutic responses, ultimately leading to personalized treatment strategies. While AI applications in genomics are traditionally associated with oncological decision-making, their integration into thoracic surgery is becoming increasingly relevant. By identifying genetic mutations (e.g., EGFR, KRAS) and predicting tumor behavior, AI enables more precise preoperative planning and risk stratification, guiding the extent of resection, lymph node dissection, or multimodal therapy selection. Moreover, gene-expression-based risk models can support intraoperative decisions and post-surgical surveillance strategies, bridging precision oncology with personalized surgical care [18,26,82].

#### 3.5.1. AI in Drug Discovery and Target Identification

You et al. explore AI’s role in anticancer drug discovery and target identification, emphasizing how biological network analysis and machine learning algorithms can map cellular interactions and uncover novel therapeutic targets. Their study highlights AI’s ability to provide a quantitative framework linking network properties to cancer progression, allowing for the identification of potential drug candidates and molecular targets. The findings contribute to the growing evidence supporting AI’s role as a game-changing tool in oncology research [18].

#### 3.5.2. Deep Learning for Gene Mutation Detection

Zhao et al. introduced DeepGEM, an AI-driven model designed to detect lung cancer gene mutations from histopathology slides, eliminating the need for traditional genomic testing. Conducted across 16 Chinese hospitals and incorporating The Cancer Genome Atlas (TCGA) data, the study aimed to develop an AI system capable of identifying mutations without human annotation. DeepGEM employed co-supervised multiple-instance learning and label disambiguation to enhance accuracy. Using a dataset of 3767 patient histology images, DeepGEM achieved exceptional accuracy for excisional biopsy samples across internal and external datasets, demonstrating strong generalizability. The model also effectively predicted EGFR and KRAS mutations in lymph node metastases, further supporting its clinical relevance. Additionally, DeepGEM generated spatial gene mutation maps, visually representing mutation distributions within tissues, positioning it as a promising tool for guiding lung cancer treatment decisions, particularly in resource-limited settings where genomic testing is inaccessible [26].

#### 3.5.3. AI-Driven Prognostic Biomarkers for Immunotherapy

Shu, Jiang, and Zhao applied ML techniques to identify a seven-gene signature predictive of lung adenocarcinoma prognosis and immunotherapy response. By analyzing single-cell and bulk RNA sequencing data, they identified 27 immune-related genes, clustering LUAD patients into three immune-infiltration-based subgroups with distinct survival outcomes. Further analysis led to the identification of seven key prognostic genes, which were integrated into a risk model that stratified patients into high- and low-risk groups. The study demonstrated that this AI-driven gene signature could predict responses to immunotherapy and chemotherapy, providing a valuable tool for personalized treatment and reinforcing AI’s potential in precision oncology [82].

### 3.6. AI in Predicting Outcomes for Infectious Thoracic Diseases

AI-driven technologies are increasingly being explored in the diagnosis, management, and treatment of infectious thoracic diseases, addressing diagnostic delays, antibiotic resistance, and treatment failure. By leveraging machine learning and deep learning models, AI can assist in early detection, risk stratification, and therapeutic decision-making, ultimately improving clinical outcomes and patient survival. While AI has been extensively studied in oncology, its potential in infectious thoracic diseases, such as thoracic empyema, parapneumonic effusions, and pleural infections, remains underrepresented and warrants further exploration [23,83,84].

#### 3.6.1. AI in Diagnosing and Managing Pleural Empyema

Zumla et al. highlight AI’s transformative potential in thoracic empyema diagnosis and treatment, particularly in improving imaging interpretation, antibiotic stewardship, and surgical outcomes. Their review discusses how machine learning models applied to CT scans and chest X-rays can detect and classify pleural effusions and empyema with high precision, often revealing complex imaging details that might be overlooked by human radiologists. AI-powered decision-support systems (DSSs) can also accelerate diagnosis, facilitate risk stratification, and optimize antibiotic use, reducing the likelihood of treatment failure and antibiotic resistance. Beyond diagnosis, AI can contribute to preoperative planning, surgical technique refinement, and long-term patient monitoring. Predictive models can assist clinicians in determining when surgical intervention is necessary, reducing unnecessary invasive procedures and ensuring early intervention in high-risk cases. These advancements may decrease hospital stays, improve patient outcomes, and streamline healthcare resource utilization. The review underscores the need for large-scale AI-driven studies to further validate these findings and integrate AI seamlessly into clinical practice [23].

#### 3.6.2. Machine Learning in Parapneumonic Effusions

In 2018, Ost explored machine learning applications in managing complicated parapneumonic effusions (CPEs), emphasizing that while AI models enhance diagnostic accuracy and treatment decisions, they must complement, rather than replace, established clinical principles. He highlights the critical role of traditional approaches, including timely drainage and appropriate antibiotic therapy, in ensuring optimal patient outcomes. The author warns against over-reliance on AI without clinical context, as this could lead to misinterpretation of results and suboptimal decision-making. Instead, he advocates for a synergistic approach, where machine learning tools support, rather than dictate, clinical decisions. This perspective highlights the importance of integrating AI with human expertise to maximize its potential in CPE management [83].

#### 3.6.3. Predicting Intrapleural Therapy Failure in Pleural Infections

Khemasuwan et al. conducted a multicenter study assessing AI’s role in predicting treatment failure in intrapleural fibrinolytic therapy using tissue plasminogen activator (tPA) and DNase for pleural infections. Their machine learning model analyzed clinical, radiographic, and laboratory data to identify key predictors of unsuccessful treatment outcomes. The model identified pleural thickening, abscess formation, and necrotizing pneumonia as significant predictors of treatment failure, suggesting that patients with these findings may not benefit from standard tPA/DNase therapy. These insights could help clinicians select alternative treatment strategies earlier, reducing unnecessary exposure to ineffective treatments and improving overall patient outcomes. Moreover, AI models could be applied to prospective studies to refine pleural infection management strategies, integrating real-time patient data to optimize clinical decision-making. By identifying high-risk patients earlier, AI could facilitate timely surgical referrals, personalized therapeutic adjustments, and enhanced long-term follow-up [84].

### 3.7. AI in Oncological Therapies

**Figure 5:**
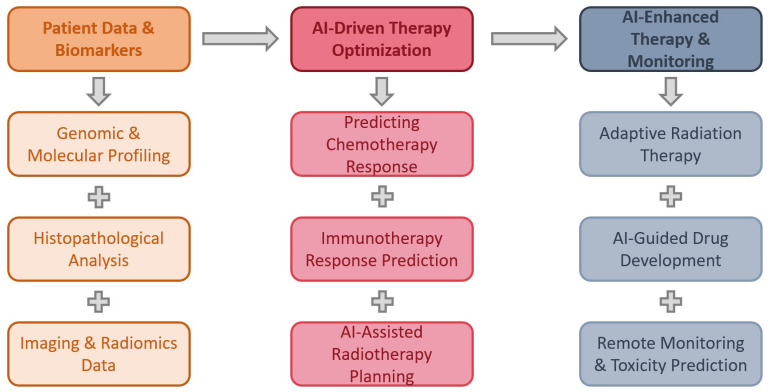
The role of AI in optimizing oncological therapies through a structured three-step process. The first step involves patient data collection and biomarker analysis, integrating genomic and molecular profiling, histopathological assessments, and radiomics-based imaging analysis. AI-driven models then facilitate therapy optimization, predicting responses to chemotherapy, immunotherapy, and radiotherapy, aiding in personalized treatment selection. Finally, AI enhances therapy administration and patient monitoring, including adaptive radiation therapy, AI-guided drug development, and remote monitoring for treatment-related toxicities. This framework enables a more precise, efficient, and personalized approach to cancer treatment, improving patient outcomes and minimizing adverse effects.

AI is revolutionizing cancer treatment strategies, particularly in chemotherapy, immunotherapy, and radiotherapy, by leveraging machine learning algorithms to optimize drug selection, minimize toxicities, and predict therapy responses. These advancements enable personalized medicine, ensuring that patients receive tailored treatments based on their unique clinical and molecular profiles. Figure 5 presents a diagram summarizing the applications of AI in oncological treatment strategies.

#### 3.7.1. AI in Chemotherapy Optimization

Torok et al. introduced an AI-driven approach for predicting chemotherapy effectiveness in lung cancer patients by analyzing serum N-glycome profiles. Their study of 33 patients utilized capillary electrophoresis with laser-induced fluorescence detection, followed by machine-learning-based classification of N-linked oligosaccharides. Structural changes in N-glycans were strongly associated with chemotherapy responses. This approach demonstrates AI’s potential to provide rapid, non-invasive insights into chemotherapy effectiveness, facilitating better treatment planning [85].

She et al. developed a deep learning model to predict major pathological response (MPR) to neoadjuvant chemoimmunotherapy in NSCLC using pretreatment CT scans from 440 patients across multiple centers. The model demonstrated high predictive accuracy, suggesting its role as a non-invasive tool for identifying patients most likely to benefit from neoadjuvant therapy, thereby enhancing personalized treatment strategies [86].

Gu and Li evaluated the efficacy of pemetrexed-platinum chemotherapy in elderly lung cancer patients using AI-enhanced CT imaging. Their study of 80 patients applied a DenseNet convolutional neural network, achieving a diagnostic accuracy of 97.4%, outperforming other AI models like LeNet-5 and ResNet. The pemetrexed group exhibited higher nursing satisfaction (92.67% vs. 85.62%) and fewer adverse reactions (fatigue, diarrhea, neutropenia) compared to the docetaxel group, demonstrating AI’s potential to optimize chemotherapy selection and patient outcomes [87].

Mei et al. investigated AI-enhanced diffusion-weighted imaging (DWI) for predicting chemotherapy-induced vomiting in early-stage lung cancer patients. Their study applied a weighted nuclear norm minimization (WNNM) algorithm to reduce noise in DWI images, improving diagnostic accuracy. The study identified apparent diffusion coefficient (ADC) values as strong predictors of chemotherapy response (sensitivity 0.95, specificity 0.89). Patients receiving AI-guided comfort care interventions experienced significantly lower vomiting rates, improved psychological well-being, and better quality of life, supporting AI’s role in enhancing supportive oncology care [88].

Lyman and Kuderer explored AI’s application in predicting febrile neutropenia (FN) risk in chemotherapy patients. Their study highlights how machine learning algorithms enhance FN risk assessment, enabling better patient stratification and prophylactic interventions. The authors emphasize the need for rigorous model validation and further research to integrate AI-based FN prediction models into clinical workflows [89].

Yang et al. reviewed AI’s role in cancer drug development, covering areas such as target validation, drug repositioning, de novo drug design, and synthetic strategy formulation. AI accelerates the drug discovery process by analyzing complex biological data, reducing development time, and improving therapeutic innovation. Despite its promise, challenges such as data standardization and interdisciplinary collaboration remain barriers to fully integrating AI in oncology drug research [90].

#### 3.7.2. AI in Immunotherapy and Targeted Therapy

AI is revolutionizing immunotherapy and targeted therapy by integrating radiomic, genomic, proteomic, and clinical data to predict treatment responses, optimize patient selection, and improve therapeutic outcomes. ML and deep learning models provide personalized treatment strategies by identifying novel biomarkers and stratifying patients based on their tumor microenvironment [91,92,93,94].

##### AI in Immunotherapy Response Prediction

Li et al. explored ML applications in lung cancer, emphasizing AI’s ability to enhance early detection, improve prognosis prediction, and personalize immunotherapy. They highlight AI’s capacity to process large, complex datasets for more accurate treatment selection, while noting challenges such as data heterogeneity and the need for validation [91].

Zhang et al. developed an AI-guided prognostic signature for lung adenocarcinoma, using 26 ML algorithms to analyze gene expression profiles. Their model stratified patients into high- and low-risk groups, correlating with survival outcomes and immunotherapy response, reinforcing AI’s role in genomic-driven personalized treatment [92].

Saad et al. introduced Deep-CT, a deep learning ensemble model predicting overall and progression-free survival in EGFR/ALK-negative NSCLC patients undergoing immune checkpoint inhibitor (ICI) therapy. By analyzing pretreatment chest CT scans from 976 patients, Deep-CT outperformed traditional risk factors (histology, smoking status, PD-L1 expression), increasing the concordance index for overall survival from 0.70 to 0.75, demonstrating AI’s predictive value in immunotherapy response [93].

Yin et al. explored AI’s multi-omics integration for predicting immunotherapy and targeted therapy outcomes. By incorporating radiomics, pathology, genomics, transcriptomics, and proteomics, AI models identified novel biomarkers beyond PD-L1 expression and oncogene mutations, offering non-invasive, highly accurate treatment predictions [94].

##### AI in Biomarker Discovery and Digital Pathology

Gao et al. reviewed AI’s role in predicting key immunotherapy biomarkers, including PD-L1, tumor mutation burden (TMB), and tumor microenvironment (TME) features. AI-driven “digital biopsy” techniques could replace or complement traditional biomarker assessments, enhancing patient stratification for immunotherapy [95].

Park et al. developed an AI-powered analyzer to classify tumor-infiltrating lymphocytes (TILs) into three immune phenotypes—inflamed, immune-excluded, and immune-desert. Patients with inflamed tumors had higher ICI response rates and longer progression-free survival, suggesting AI’s role in immune profiling for therapy optimization [96].

Cheng et al. created an AI-based deep learning model to automate PD-L1 expression analysis in 1288 lung cancer patients. The model demonstrated 96.4% accuracy and 96.8% specificity in the PD-L1 (SP263) assay, performing on par with expert pathologists, supporting AI’s role in automating and standardizing biomarker evaluation [97].

##### Radiomics and AI for Immunotherapy Precision

Roisman et al. reviewed radiomics-driven AI models for personalized immunotherapy prediction in lung cancer. They propose that AI can analyze molecular profiles, tumor resistance mechanisms, and microenvironment complexities, enhancing treatment precision and clinical decision-making [98]

Mei et al. examined the limitations of PD-L1 as a biomarker in NSCLC immunotherapy prediction, highlighting that some low-PD-L1 patients respond well to treatment. They advocate for multi-omics AI models, incorporating genomic, transcriptomic, proteomic, microbiomic, and radiomic data to refine patient stratification and treatment selection [99].

#### 3.7.3. AI in Radiotherapy

AI is playing a crucial role in enhancing radiotherapy planning, prognostication, and treatment personalization in lung cancer. By integrating radiomics, deep learning, and artificial neural networks (ANNs), AI improves risk assessment, treatment precision, and outcome prediction for patients undergoing stereotactic body radiation therapy (SBRT), stereotactic ablative radiotherapy (SABR), and postoperative radiotherapy (PORT) [100,101,102,103,104].

##### AI for Prognostic Prediction in Radiotherapy

Walls et al. conducted a systematic review on radiomics-based outcome prediction in lung cancer patients undergoing radical radiotherapy, with or without chemotherapy. Their analysis of 44 studies identified imaging texture features (e.g., gray-level run-length matrix, gray-level co-occurrence matrix, kurtosis) as significant predictors of local control, distant metastasis, overall survival (OS), and pulmonary toxicity. Despite promising results, the review highlighted methodological variability, stressing the need for standardization in radiomic analyses to improve clinical applicability [100].

Xing et al. performed a meta-analysis on AI models for radiotherapy outcome prediction in 4719 lung cancer patients across 18 studies. AI-identified high-risk patients had significantly poorer outcomes, with pooled hazard ratios (HRs) for OS, local control (LC), progression-free survival (PFS), and disease-free survival (DFS) of 2.55, 2.45, 3.84, and 2.66, respectively. The combined area under the curve (AUC) for OS (0.75) and LC (0.80) demonstrated moderate predictive accuracy. The study concluded that while AI models show promise, large-scale prospective validation studies are needed [101].

Borghetti et al. developed an AI-enhanced predictive model for OS in stage I NSCLC patients undergoing SBRT. Using Cox regression (CR) analysis and an artificial neural network (ANN), they stratified patients into two prognostic groups, with distinct OS trends across different cohorts. The model demonstrated clinical utility in patient stratification, reinforcing AI’s role in refining SBRT candidate selection [102].

##### AI in Radiation-Induced Toxicity Prediction

Chan et al. examined the impact of cardiac radiation doses on OS in early-stage NSCLC patients undergoing SABR. Their study found that higher right ventricle (RV) radiation exposure (V10Gy > 4%) significantly reduced median overall survival (2.4 vs. 5.3 years). ANN models incorporating 74 input features, including cardiac dosimetry parameters, predicted survival with 64.7% accuracy, highlighting AI’s role in assessing radiation toxicity and guiding cardiac-sparing strategies [103].

Kim et al. validated a CT-based deep learning model for predicting SABR outcomes in lung cancer. Their findings confirmed that AI-assisted models effectively stratify patients based on prognosis, demonstrating clinical applicability for personalized treatment planning [104].

##### AI-Assisted Contouring in Radiotherapy Planning

Han et al. conducted a multicenter prospective cohort study evaluating an AI-assisted auto-segmentation model for delineating clinical target volumes (CTVs) and organs at risk (OARs) in postoperative radiotherapy (PORT) for lung cancer. Their study of 55 patients compared three contouring approaches: (i) unmodified AI auto-segmentation, (ii) fully manual delineation by junior radiation oncologists, and (iii) AI-assisted manual modifications. The AI-assisted method outperformed both unmodified AI and fully manual delineation, achieving superior Hausdorff distance (HD), mean distance of agreement (MDA), and Dice similarity coefficient (DSC) values. It also reduced contouring time by 43.2% for CTVs and 56.2% for OARs, enhancing both accuracy and workflow efficiency. These findings support AI-assisted segmentation as a valuable tool for optimizing radiotherapy planning and improving treatment outcomes [105].

### 3.8. AI-Driven Robotic-Assisted Techniques in Thoracic Surgery

**Figure 6:**
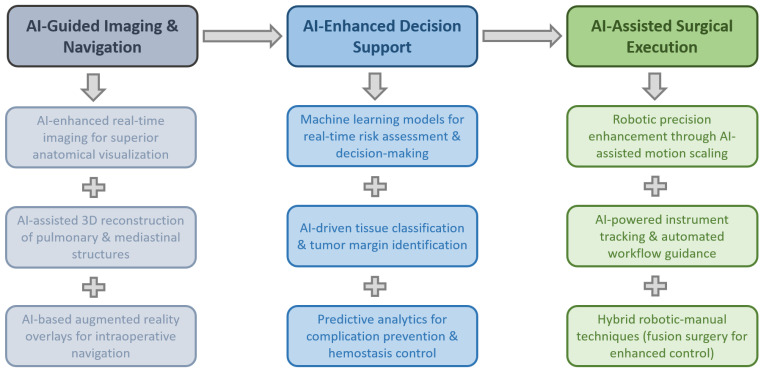
The intraoperative role of AI in robotic-assisted thoracic surgery, illustrating AI’s impact on imaging, decision support, and surgical execution. AI enhances intraoperative imaging through real-time augmented visualization and 3D reconstruction. In decision support, AI provides predictive analytics, real-time risk assessment, and tumor margin identification. Finally, AI refines surgical execution by improving robotic precision, instrument tracking, and hybrid robotic–manual techniques, contributing to greater accuracy and patient safety.

Robotic-assisted thoracic surgery has emerged as a key innovation in minimally invasive thoracic procedures, enhancing surgical precision, reducing complications, and improving patient outcomes. The integration of advanced robotic platforms has expanded the feasibility of complex thoracic surgeries, enabling better visualization, refined instrumentation, and improved ergonomics for surgeons. While AI-driven technologies hold potential for future advancements in intraoperative guidance and automation, current robotic systems primarily rely on surgeon-controlled mechanisms rather than fully AI-powered automation [35,36,37,38,39]. Figure 6 attempts to illustrate the potential intraoperative role of AI in robotic-assisted thoracic surgery

#### 3.8.1. The Increasing Role of Robotic-Assisted Techniques in Thoracic Surgery

##### Robotic Thoracic Day Surgery and Enhanced Recovery Protocols

Li et al. conducted a retrospective study comparing robot-assisted thoracic day surgery with traditional robot-assisted thoracic surgery, analyzing perioperative outcomes, safety, and efficiency. The study confirmed that robotic-assisted day surgery, when combined with enhanced recovery after surgery (ERAS) protocols, led to shorter hospital stays, reduced drainage times, and lower overall medical costs, while maintaining comparable complication rates to traditional robotic surgery. The findings support the growing role of robotic techniques in optimizing efficiency and resource utilization in thoracic surgery [35].

##### Robotic Lobectomy: Expanding Adoption and Optimizing Patient Outcomes

Fairbairn et al. highlighted the significant increase in robotic lobectomy adoption in the US over the past decade, driven by improved perioperative outcomes and patient preference for minimally invasive approaches. Compared to VATS, robotic lobectomy provides greater instrument precision, better maneuverability, and enhanced 3D visualization. The study also mentions the importance of surgeon expertise and standardized training programs to ensure the safe and effective integration of robotic techniques in clinical practice [36].

##### Evolution of Robotic Thoracic Surgery and Technological Milestones

Lazar and Hwalek reviewed the historical progression of robotic thoracic surgery, detailing innovations such as improved robotic instrument articulation, 3D high-definition imaging, and better haptic feedback mechanisms. The study emphasizes that while AI-driven autonomous surgical systems remain in early development, ongoing advancements in robotic platforms and real-time intraoperative navigation systems are likely to further enhance surgical precision. The future may see augmented reality (AR) integration and machine-learning-based intraoperative decision support as part of next-generation robotic surgical systems [37].

##### Robotic Surgery for Mediastinal Disease

Seastedt et al. explored the application of robotic surgery in mediastinal procedures, particularly for thymectomy and mediastinal mass resections. The study found that robotic-assisted mediastinal surgery offers superior visualization and instrument control compared to conventional sternotomy, resulting in reduced invasiveness, faster recovery, and improved surgical outcomes. The findings reinforce the growing role of robotic techniques in complex mediastinal procedures, though surgeon expertise and case selection remain critical factors [38].

##### Challenges and Learning Curve in Robotic Thoracic Surgery

Veronesi examined the technical learning curve associated with robotic thoracic surgery, particularly in pulmonary resections for lung cancer. While robotic platforms enhance visualization and dexterity, widespread adoption is limited by high costs, lengthy operative times, and a need for specialized training. The study suggests that increasing competition among robotic surgical companies may drive cost reductions, making robotic platforms more accessible. Future research is needed to evaluate long-term patient outcomes, quality of life, and oncologic radicality compared to other surgical approaches [39].

##### Perioperative Considerations in Robotic Thoracic Surgery

Steenwyk and Lyerly highlighted the perioperative challenges associated with robotic-assisted thoracic surgery, particularly for anesthesia management. Due to longer operative times, single-lung ventilation requirements, and unique patient positioning constraints, robotic surgery presents distinct anesthetic challenges. The study underscores the need for careful intraoperative monitoring, ventilation optimization, and hemodynamic management to ensure safe patient outcomes during robotic-assisted thoracic procedures [40].

##### Hybrid “Fusion Surgery”: Combining Robotic Assistance with Manual Techniques

Tane et al. introduced the concept of fusion surgery, a hybrid approach that combines robotic assistance with manual intraoperative techniques, addressing limitations such as a lack of tactile feedback and steep learning curves. In this technique, the table surgeon performs key tasks such as lung retraction and stapling, while the robot handles fine dissection and tissue manipulation. The study suggests that hybrid approaches may serve as a transitional strategy, allowing surgeons to gradually adapt to robotic platforms while retaining critical tactile feedback mechanisms [41].

##### Institutional Experience in Scaling Robotic Surgery Programs

Herrera et al. analyzed the implementation of a RATS program over eight years, reviewing 2500 cases. The study demonstrated that with experience and technical mastery, surgical times and hospital stays decreased, supporting the long-term benefits of robotic surgery in thoracic procedures. The findings highlight the importance of structured training programs, resource allocation, and continuous data collection in establishing successful robotic surgery programs [43].

##### Future Directions and Research in Robotic Thoracic Surgery

Wang’s bibliometric analysis examined global research trends in robotic thoracic surgery, identifying key topics such as lung cancer resections, esophageal surgery, and minimally invasive techniques. The study noted an increasing focus on augmented reality-assisted navigation, deep learning-based surgical analytics, and robotic automation. Although AI-driven robotic surgery remains in early development, future advancements in machine learning for intraoperative guidance and autonomous surgical systems may further optimize robotic-assisted thoracic procedures [44].

##### Building Sustainable Robotic Surgery Programs

Hompe, Furlow, and Schumacher provided a strategic framework for institutions seeking to establish robotic thoracic surgery programs, emphasizing administrative support, multidisciplinary collaboration, and surgeon training. They discussed key considerations such as cost-effectiveness, patient selection, and integration into existing surgical workflows. The study concludes that carefully planned robotic programs can enhance patient outcomes and advance institutional surgical capabilities, ensuring long-term sustainability and financial viability [106].

#### 3.8.2. Implications of AI in Intraoperative Support

AI is increasingly playing a critical role in intraoperative decision support, real-time surgical navigation, and anatomical reconstruction in thoracic surgery. AI-driven 3D reconstruction, machine-learning-based skill assessment, and predictive analytics are improving surgical precision, efficiency, and patient safety, while also reducing operation times and intraoperative complications [42,107].

##### AI-Based 3D Reconstruction

Li et al. conducted a comprehensive study evaluating an AI-driven three-dimensional (3D) reconstruction system designed to enhance thoracic surgical planning and intraoperative navigation. The system, based on a 3D convolutional neural network trained on 500 cases, achieved high anatomical prediction accuracy, with a Dice coefficient of 89.2%, significantly outperforming manual reconstruction techniques. The AI system automatically reconstructed pulmonary broncho-vascular structures, reducing reliance on manual segmentation and human interpretation errors [107].

The study assessed prospective and retrospective cohorts undergoing segmentectomies or lobectomies, comparing AI-generated digital reconstructions with actual anatomical structures observed intraoperatively. The AI model demonstrated remarkable accuracy, correctly identifying segmental bronchi in 100% of retrospective cases and 97% of prospective cases, while segmental veins and arteries were detected with over 93% accuracy. Additionally, when compared to manual reconstruction software (Mimics^®^,MIS 24.0, Leuven, Belgium, Materialize), the AI model reduced the average model reconstruction time by 14.2 min, while producing higher-quality 3D models. Clinically, the use of AI reduced surgical time by 24.5 min for lobectomies and 20 min for segmentectomies, demonstrating its potential to streamline surgical workflows and improve operating room efficiency. Safety assessments, including intraoperative blood loss and complication rates, confirmed that AI-assisted surgical planning led to improved outcomes without compromising safety. These findings validate the practical applicability of AI in intraoperative guidance, suggesting that AI-assisted 3D reconstruction could become a standard tool in modern thoracic surgery [107].

##### Machine Learning in Intraoperative Decision Support and Surgical Skill Assessment

Ostberg et al. explored how ML is transforming thoracic surgery, emphasizing its role in data-driven surgical decision-making, skill evaluation, and predictive analytics [42]. The study highlighted five key areas where ML can impact thoracic surgery:

1. Enhanced Diagnosis—AI-driven image analysis and clinical data interpretation can improve early disease detection and intraoperative navigation, leading to better surgical decision-making.
2. Surgical Skill Assessment—ML models can provide real-time performance reviews, offering personalized feedback to surgeons for skill enhancement and optimized outcomes.
3. Postoperative Prognostication—AI-powered risk prediction models can assess individualized patient recovery trajectories, allowing for tailored postoperative care strategies.
4. Intraoperative Performance Optimization—ML algorithms can provide real-time analysis of patient data, alerting surgeons to potential complications or deviations from optimal surgical techniques.
5. Accelerating Translational Research—AI can analyze large-scale surgical datasets, identifying patterns that inform new techniques, improve surgical efficiency, and develop novel therapeutic approaches.

While ML offers unprecedented advancements, the study also highlighted several limitations. One major challenge is the “black box” nature of ML models, making it difficult to interpret algorithmic decision-making in surgical environments. Additionally, data limitations—including insufficient high-quality clinical datasets—pose a barrier to developing fully reliable and generalizable AI models. Ethical concerns such as patient privacy, algorithmic bias, and data security must also be addressed to ensure trustworthy AI integration into surgical practice [42].

#### 3.8.3. Augmented Cognition and Computer Vision in the Operating Room

Technological advancements have revolutionized the operating room (OR), transforming it into a data-driven environment where AI enhances surgical workflows, decision-making, and team dynamics. AI systems process real-time intraoperative data to support cognitive functions, improving situational awareness, coordination, and problem-solving in high-stakes surgical procedures. By predicting future surgical events and adapting to evolving conditions, AI assists surgical teams in optimizing performance throughout complex operations [108,109,110].

AI-driven cognitive monitoring employs physiological indicators such as heart rate variability (HRV), electroencephalography (EEG), and near-infrared spectroscopy (NIRS) to assess surgeons’ cognitive load and mental fatigue. These tools enable context-aware AI assistance, ensuring that surgeons and OR teams operate at peak efficiency while minimizing cognitive overload. By tracking these physiological parameters, AI enhances team synchronization, workload distribution, and adaptive decision-making, contributing to a safer and more efficient surgical environment [108,109].

A key AI-driven innovation in the OR is computer vision (CV), which processes visual data from intraoperative imaging and video feeds to support real-time surgical navigation and technical skill assessment. In thoracic and cardiothoracic surgery, CV is applied for workflow segmentation, surgical instrument recognition, and movement tracking, allowing AI to analyze surgeon precision, team interactions, and procedural efficiency. This capability enables objective skill evaluation, procedural standardization, and performance optimization, advancing the concept of cognitive surgery—where AI enhances both technical execution and cognitive processing [111,112,113].

By integrating AI-powered augmented cognition and computer vision, the OR becomes a collaborative ecosystem where technology enhances both individual and team performance. These innovations contribute to greater precision, improved communication, and enhanced decision-making, ultimately redefining the landscape of high-complexity surgeries, including thoracic and cardiothoracic procedures [109].

### 3.9. Postoperative Care and Follow-Up in Thoracic Surgery

AI is transforming postoperative management in thoracic surgery by enabling real-time patient monitoring, early detection of complications, and predictive analytics for personalized recovery plans. AI-powered tools facilitate proactive decision-making, improving long-term follow-up strategies and overall quality of life. By integrating machine learning algorithms, wearable monitoring devices, and AI-assisted imaging, postoperative care is becoming more efficient, patient-centered, and data-driven.

#### 3.9.1. AI-Assisted Postoperative Recovery in Robotic Thoracic Surgery

Hu and He conducted a study evaluating postoperative outcomes following robot-assisted thoracic surgery using the Da Vinci system (Da Vinci Robotic Surgical Inc., Intuitive Surgical Inc. and Mountain View Intuitive), focusing on its impact on recovery, safety, and surgical precision. The study included 42 patients who underwent robot-assisted lobectomy between January and December 2014 [34]. The findings demonstrated favorable intraoperative and postoperative outcomes, including minimal blood loss, stable vital signs, and operation times ranging from 62 to 225 min. None of the patients required intraoperative blood transfusions, and two patients underwent robotic wedge resection followed by robotic lobectomy after intraoperative malignancy confirmation, without any cases requiring conversion to thoracotomy.

Postoperatively, patients reported minimal discomfort, had no infections, and exhibited optimal wound healing, enabling early mobilization and shorter hospital stays. The study highlighted the benefits of AI-assisted robotic surgery, such as reduced surgical trauma, lower postoperative pain, enhanced precision in lymph node dissection, and faster recovery. These results support the growing role of AI-powered robotic systems in thoracic surgery, demonstrating their potential to improve surgical accuracy and optimize patient outcomes.

#### 3.9.2. AI-Enhanced Lung Ultrasound for Postoperative Monitoring

Malík et al. conducted a systematic review exploring the role of lung ultrasound (LUS) as an alternative to chest X-rays (CXRs) in postoperative monitoring following thoracic surgery [114]. Analyzing ten studies, the review found that eight studies demonstrated that LUS could effectively reduce CXR dependency without compromising patient safety.

The authors also discussed the emerging role of AI in enhancing LUS applications, suggesting that AI could be used for the following:

- Improving diagnostic accuracy by detecting postoperative complications such as pleural effusions, pneumothorax, and atelectasis with greater precision.
- Standardizing ultrasound interpretations, reducing interobserver variability and improving diagnostic consistency.
- Accelerating learning curves, making LUS a more accessible tool for thoracic surgeons and critical care teams.
- Minimizing inconclusive results, ensuring that imaging-based decisions are more reliable and reproducible.

The study advocates for integrating AI-assisted LUS into standard postoperative protocols, emphasizing the need for clinical trials to validate AI’s potential in optimizing thoracic surgery follow-up care.

#### 3.9.3. AI in Predicting Postoperative Complications and Remote Patient Monitoring

A previous study explored the role of AI in postoperative care for cardiac surgery, highlighting its potential to predict complications, improve patient monitoring, and reduce healthcare costs [109]. These findings are equally relevant to thoracic postoperative management, as AI-powered systems can accomplish the following:

- Analyzing patient data to forecast postoperative risks such as pulmonary infections, delayed recovery, and respiratory complications, enabling timely medical interventions.
- Facilitating continuous remote monitoring through wearable devices and AI-driven telemedicine platforms, ensuring early detection of adverse events and reducing hospital readmissions.
- Optimizing resource allocation in hospitals by providing real-time recovery predictions, allowing personalized follow-up strategies tailored to each patient’s needs.

Despite these advantages, several challenges remain, including data quality, model validation, ethical considerations, and clinical workflow integration. Addressing these issues is crucial to widespread AI adoption in postoperative thoracic surgery, ensuring that AI-driven innovations translate into meaningful clinical improvements.

### 3.10. AI in Education and Research in Thoracic Surgery

AI is revolutionizing thoracic surgery education and research by enhancing training methodologies, predictive modeling, and virtual simulations. AI-driven platforms facilitate skill development, real-time feedback, and data-driven research advancements, fostering innovation and continuous learning in the field.

#### 3.10.1. AI in Surgical Training and Skill Development

Mitzman et al. emphasized the need for standardized training in robotic thoracic surgery, highlighting the role of simulation and video-based coaching in skill acquisition. As robotic platforms become increasingly prevalent, structured training programs help residents refine technical abilities, build confidence, and improve efficiency, ultimately contributing to better patient outcomes [32].

Raad et al. addressed the lack of formalized robotic-assisted thoracic surgery training in residency programs, proposing a structured curriculum spanning postgraduate years 2 to 6 [115]. Their model incorporates online modules, virtual reality simulations, in-house workshops, and progressive clinical exposure—starting with bedside assistance and culminating in console-based procedures. This stepwise approach ensures that residents gain proficiency before entering independent practice.

Sachdeva and Sethi explored AI’s impact on bronchoscopy training, citing studies where AI-driven feedback significantly improved technical skills and increased trainee engagement [116]. AI-assisted training modules provided real-time guidance, reducing errors and enhancing performance in simulated environments. Similarly, Cold et al. conducted a randomized controlled trial demonstrating that AI-based bronchial segment identification systems enhanced bronchoscopy proficiency in novices. Compared to traditional instruction, AI feedback led to faster procedure times, improved navigation accuracy, and greater diagnostic completeness, supporting its potential for broader implementation in clinical education [45].

Beyond enhancing technical skill acquisition, AI is increasingly integrated into simulation-based surgical education through high-fidelity virtual reality (VR) and augmented reality (AR) environments. These platforms replicate complex thoracic procedures in immersive, risk-free settings, enabling trainees to repeatedly practice critical tasks without endangering patients. AI algorithms within these simulators provide real-time, objective feedback on instrument handling, procedural accuracy, and decision-making, thereby facilitating personalized, adaptive learning. Moreover, machine learning models can track a trainee’s performance over time, identifying strengths and weaknesses and adjusting the difficulty of scenarios accordingly. AI-powered video analysis and motion tracking tools are also being employed to assess intraoperative technique, surgical efficiency, and adherence to procedural protocols. These technologies support the transition toward competency-based education by offering standardized, data-driven evaluations of surgical skill. As minimally invasive and robotic-assisted procedures continue to expand, the integration of AI into surgical training will be pivotal for preparing the next generation of thoracic surgeons with both technical proficiency and clinical confidence [45,115,116].

#### 3.10.2. AI in Medical Education and Exam Performance

Gencer and Aydin evaluated ChatGPT’s (OpenAI, San Francisco, CA, USA) potential in thoracic surgery education, comparing its performance on 105 medical exam questions against medical students. While students scored 83.33% on average, ChatGPT-3.5 achieved 90.48%, and ChatGPT-4 outperformed both at 93.33% [48]. These results suggest that large language models (LLMs) can complement medical training, providing instant explanations, answering complex queries, and serving as interactive learning tools.

#### 3.10.3. AI-Driven Research and Predictive Analytics

AI-powered research platforms are accelerating scientific discovery in thoracic surgery, leveraging machine learning algorithms to analyze vast datasets, identify patterns, and predict clinical outcomes. As AI becomes more integrated into training and research, its potential to enhance education, refine surgical techniques, and personalize learning experiences continues to grow. However, challenges remain in standardizing AI-driven training, ensuring ethical implementation, and validating AI-assisted assessments for widespread adoption [45,48].

A summary of selected key studies, AI modalities, and reported performance metrics is presented in Table 3. Given the methodological heterogeneity, we synthesized performance data descriptively, highlighting the clinical relevance of AI applications across diagnostic, surgical, and prognostic domains.

## 4. Discussion

This review highlights the transformative role of AI in thoracic surgery, particularly in preoperative planning, intraoperative assistance, and postoperative management. AI enhances risk stratification, surgical precision, and patient monitoring, ultimately contributing to improved clinical outcomes and efficiency [84,91]. However, several challenges remain, including data integration, ethical concerns, cost constraints, and implementation barriers [114,117]. To maximize AI’s benefits while ensuring safety, reliability, and widespread adoption, further systematic research, validation, and interdisciplinary collaboration are essential.

### 4.1. AI and Robotic-Assisted Thoracic Surgery: Opportunities and Challenges

The increasing body of literature supports the growing use of RATS, demonstrating advantages over traditional open surgery and VATS [35,36,37]. RATS offers greater surgical precision, enhanced visualization, improved ergonomics, and better postoperative recovery times. Studies evaluating robotic lobectomies, minimally invasive esophagectomies, and lung cancer resections consistently report faster recovery periods, lower complication rates, and comparable—if not superior—oncologic outcomes [38,39,40,41].

Platforms such as the Da Vinci surgical system improve surgical efficiency by reducing operating times and optimizing lymph node dissection, leading to better long-term prognoses [34,43]. However, widespread implementation faces several obstacles:

- High upfront costs associated with robotic platforms pose a financial barrier, particularly in low-resource healthcare settings [114].
- A steep learning curve requires specialized training and credentialing, delaying the adoption of robotic-assisted techniques [115].
- Limited accessibility in certain institutions due to a lack of infrastructure and high maintenance costs [109].

Current research suggests that in high-volume centers, the long-term benefits of RATS—such as fewer complications, shorter hospital stays, and enhanced functional recovery—may outweigh the initial investment [32,44]. To mitigate learning curve challenges, standardized training protocols, credentialing pathways, and inter-institutional collaborations should be prioritized [115].

### 4.2. AI’s Role in Diagnostics, Perioperative Care, and Prognostic Modeling

Etienne et al. provide a comprehensive analysis of AI applications in thoracic surgery, exploring its role in diagnostics, prognostic decision-making, and intraoperative assistance [11]. AI is increasingly utilized for early lung cancer detection, perioperative risk assessment, and outcome prediction, particularly through radiomics-based imaging analysis, histopathology models, and genomic profiling tools [57,64,82]. AI-powered imaging interpretation, pathology analysis, and clinical decision-support systems are enhancing the accuracy and efficiency of thoracic surgery [10,48,62].

However, AI adoption in thoracic surgery remains limited compared to other medical specialties, largely due to regulatory, ethical, and technical challenges [117]. In European healthcare systems, concerns regarding AI-driven clinical decision-making, liability issues, and patient data privacy regulations pose significant barriers [115]. To bridge the gap between AI potential and real-world implementation, surgeons must develop a foundational understanding of AI’s capabilities, fostering collaborations between medical professionals, data scientists, and regulatory bodies [114].

### 4.3. AI in Perioperative Management and Postoperative Monitoring

Aleem et al. highlight AI’s role in perioperative management, particularly in electronic medical record extraction, automated risk assessment, and real-time monitoring [118]. AI-driven ML models enhance preoperative patient stratification, improving surgical decision-making [24]. Additionally, AI-powered natural language processing (NLP) systems facilitate automated clinical documentation, workflow optimization, and early complication detection [48,69].

Despite these advancements, several challenges persist:

- Algorithmic bias—AI models trained on limited or non-diverse datasets may fail to generalize across different patient populations [61,62].
- Data security risks—AI’s reliance on large-scale patient data raises concerns regarding privacy, cybersecurity, and compliance with healthcare regulations [109].
- Ethical considerations—Automated decision-making must maintain transparency, interpretability, and alignment with medical ethics [117].

To address these concerns, future studies should focus on AI explainability, dataset diversity, and ethical AI governance, ensuring safe and unbiased AI applications in surgical practice [114].

### 4.4. Standardizing AI Integration into Thoracic Surgery

Herrmann, Oggiano, and Hecker discuss the current limitations of AI in thoracic surgery, emphasizing that AI is primarily applied in diagnostics and therapy planning but lacks full integration into surgical workflows. They note that while AI-powered computer-aided diagnostic tools and robotic-assisted surgical systems have shown promise, fully autonomous AI-driven surgery remains a distant goal [117].

For broader AI adoption, standardized data collection, structured validation frameworks, and clinical trials are essential. Additionally, clear ethical guidelines, legal liability frameworks, and interdisciplinary cooperation must be established to promote responsible AI integration in thoracic surgery [114].

A critical limitation in the current body of evidence is the lack of large-scale, multicenter prospective studies aimed at validating the clinical efficacy and real-world applicability of AI tools in thoracic surgery. Although numerous retrospective and single-center studies report encouraging results, their findings often suffer from limited external validity due to homogeneous patient populations, constrained institutional settings, and varying clinical protocols. This lack of diverse and representative datasets can lead to the overfitting of AI models and reduced performance when AI models are deployed in broader clinical environments. Furthermore, many studies focus primarily on technical performance metrics, such as accuracy or AUC, without evaluating meaningful clinical endpoints like complication rates, operative efficiency, or long-term patient outcomes. To move beyond a proof of concept and ensure safe, equitable, and effective integration of AI into thoracic surgical practice, there is an urgent need for prospective trials that are multicentric in design, incorporate standardized methodologies for AI evaluation, and align with regulatory and ethical frameworks. Such studies should aim to assess not only the performance of AI algorithms but also their impact on clinical decision-making, workflow optimization, patient safety, and cost-effectiveness. Only through rigorous clinical validation can AI-based systems gain the trust of healthcare professionals and regulatory bodies, facilitating their transition from experimental tools to standard components of surgical care.

### 4.5. Real-World Barriers: Economic, Regulatory, Ethical, and Implementation Challenges

While the technical potential of AI in thoracic surgery is well documented, its real-world adoption remains limited due to several practical challenges that extend beyond algorithmic performance and is constrained by a combination of technical, institutional, and human factors. These include substantial financial, infrastructural, and regulatory barriers that must be addressed to facilitate the clinical integration of AI-based tools [118].

A critical challenge is the high economic cost associated with AI development, deployment, and maintenance. The need for robust computing infrastructure, annotated datasets, continuous algorithm training, and interdisciplinary collaboration imposes significant financial burdens, particularly on smaller healthcare institutions or those in resource-limited settings. Additionally, successful integration requires investment in personnel training and workflow restructuring, which can further delay implementation and add to the total cost of ownership [119].

From a regulatory perspective, current approval pathways for AI-based tools are still evolving. In the United States, the Food and Drug Administration (FDA) has introduced frameworks such as Software as a Medical Device (SaMD) and is developing guidelines for adaptive algorithms. However, continuous-learning AI models pose regulatory challenges due to their dynamic nature [120,121]. Similarly, within the European Union, the European Medicines Agency (EMA) and national authorities are working to accommodate AI under the new Medical Device Regulation (MDR), but comprehensive, harmonized frameworks for evaluating safety, efficacy, and data security are still lacking [122,123]. Transparency, reproducibility, and ongoing post-market surveillance are emphasized by both agencies, yet specific guidelines for explainable or adaptive AI remain underdeveloped.

Another major barrier is the lack of interoperability between AI systems and existing hospital information infrastructures, which hinders seamless data exchange and integration into clinical workflows. Additionally, the limited availability of user-friendly interfaces and the steep learning curve associated with AI technologies contribute to clinician resistance, especially in high-stakes environments like the operating room. The successful integration of AI also requires alignment with institutional priorities, availability of technical support, and consistent performance in dynamic, time-sensitive surgical settings—criteria that are not yet fully met by many current AI applications [124,125].

Alongside logistical challenges, ethical and regulatory concerns represent substantial hurdles. Patient data privacy remains a critical issue, particularly given the volume and sensitivity of clinical and imaging data required to train AI models. Even when datasets are anonymized, the potential for re-identification persists, especially when combined with external databases. Algorithmic bias, introduced through imbalanced or non-representative training data, poses a significant risk of exacerbating existing health disparities. Moreover, the opacity of many AI models—often referred to as “black box” systems—can undermine clinical trust and hinder accountability in decision-making [126,127]. Regulatory agencies such as the FDA and EMA are working to establish guidelines for AI oversight, including frameworks for continuous learning systems and post-deployment monitoring, but challenges remain [120,121].

Overcoming these economic and regulatory barriers is essential for translating the promise of AI into tangible clinical benefits. Coordinated efforts among policymakers, regulatory bodies, clinicians, ethicists, and developers will be key to ensuring that AI systems are not only technically advanced but also financially feasible, ethically sound, and operationally sustainable within real-world surgical environments. A responsible path forward will require close collaboration among these partners, to ensure that AI systems are safe, equitable, explainable, and ethically aligned with patient-centered care.

## 5. Future Perspectives

The future of AI in the thoracic surgery specialty is expected to be shaped by emerging technologies such as digital twins, deep learning, natural language processing, and federated learning. These advancements hold the potential to optimize surgical decision-making, enhance precision, and improve long-term patient outcomes. However, for AI to be effectively and responsibly integrated into thoracic surgery, critical challenges must be addressed, including ethical concerns, data security, regulatory frameworks, and interdisciplinary collaboration.

### 5.1. Advancing AI in Thoracic Surgery

Jones et al. discuss the transformative role of AI in cardiothoracic surgery, emphasizing how ML surpasses traditional predictive models like logistic regression by leveraging deep CNNs to analyze thousands of clinical variables simultaneously [30,49]. AI’s ability to detect complex patterns and correlations has the potential to refine clinical decision-making, optimize surgical planning, and reduce diagnostic errors. However, the authors caution that while AI can identify statistically significant predictors, it may still produce misleading or irrelevant outputs, reinforcing the need for continuous human oversight and validation in AI-driven medical applications.

The integration of AI into thoracic surgery spans multiple domains, from enhancing precision medicine in genetic research to refining diagnostic accuracy in lung cancer detection and surgical planning [11,30]. Deep learning algorithms can automatically segment pulmonary nodules, analyze tumor microenvironments, and assess lymph node metastases, providing more precise prognostic insights and individualized treatment recommendations [56,93,96]. Additionally, AI-powered decision-support systems can assist in selecting optimal surgical strategies based on patient-specific risk factors, contributing to personalized and data-driven healthcare.

### 5.2. Ethical and Practical Challenges in AI Implementation

Despite rapid technological advancements, several barriers remain in the widespread clinical adoption of AI in thoracic surgery:

- Algorithmic Bias—AI models trained on non-diverse datasets may result in biased predictions, disproportionately affecting underrepresented patient populations [61,62]. Ensuring algorithmic fairness requires multi-institutional, diverse training datasets and ongoing bias assessment in AI models.
- Data Security and Privacy—AI’s reliance on large-scale patient data raises concerns regarding confidentiality, cybersecurity, and regulatory compliance [109]. Implementing federated learning approaches, where AI models are trained across multiple institutions without sharing raw data, may help mitigate privacy risks while preserving data integrity.
- Regulatory and Legal Considerations—Clear guidelines for AI validation, liability, and clinical responsibility must be established before autonomous AI systems can be fully trusted in surgical settings [117]. Policymakers must develop legal frameworks to address AI-driven clinical decision-making and define accountability measures for AI errors or misdiagnoses.
- Integration into Clinical Practice—AI-driven solutions must be seamlessly incorporated into existing surgical workflows, requiring user-friendly interfaces, clinician training, and robust clinical validation [49]. AI tools should function as augmentative systems, complementing rather than replacing surgeon expertise.

### 5.3. Further Expanding AI’s Role in Thoracic Surgery

To fully unlock AI’s potential, future research should prioritize the following:

- Digital Twin Technology—creating virtual patient models to simulate surgical outcomes, predict complications, and personalize treatment planning based on real-time physiological data.
- Explainable AI (XAI)—developing transparent AI algorithms that provide interpretable, clinician-friendly insights to enhance trust and adoption in surgical settings.
- Multicenter AI Validation—conducting large-scale, multi-institutional trials to assess the generalizability and clinical utility of AI models across diverse patient populations and healthcare systems.
- Autonomous Surgical Assistance—advancing AI-powered robotic platforms capable of providing real-time intraoperative decision support, including predictive analytics for bleeding risks, automated suturing, and augmented visualization through AI-enhanced imaging systems.
- AI-Augmented Training Programs—implementing AI-driven virtual reality (VR) and simulation-based training modules to accelerate surgeon proficiency in robotic-assisted techniques while ensuring standardized, high-quality education.
- Integration of Multi-Omics Data—combining genomic, proteomic, radiomic, and clinical data to develop comprehensive AI models for thoracic oncology, improving precision in diagnosis, prognosis, and treatment selection.

As AI continues to evolve and integrate with robotic surgery, precision medicine, and real-time clinical decision support, it holds the potential to revolutionize thoracic surgery, enhancing both patient care and surgical efficiency. However, responsible implementation, ethical governance, and continuous validation remain essential prerequisites for AI’s widespread adoption in thoracic surgery.

## 6. Limitations

While this review highlights the growing impact of artificial intelligence (AI) in thoracic surgery, several limitations must be acknowledged.

Heterogeneity in AI Applications and Study Designs: The wide variability in AI models, datasets, and evaluation metrics among studies makes it difficult to directly compare results or establish standardized benchmarks for AI performance in thoracic surgery. Differences in training datasets, algorithm architectures, and validation methods contribute to inconsistencies in reported outcomes, limiting the generalizability of findings across different institutions and patient populations.

Lack of Large-Scale Prospective Clinical Trials: Most AI applications in thoracic surgery have been evaluated in retrospective or single-center studies, with limited prospective, multicenter randomized controlled trials (RCTs) conducted to validate their real-world effectiveness. The lack of high-quality, large-scale validation restricts the clinical translation of AI-driven tools and highlights the need for more rigorous, externally validated studies.

Algorithmic Bias and Data Representation Challenges: AI models rely on large datasets, and many current AI-driven tools have been trained on non-diverse or geographically limited datasets, leading to algorithmic biases that may disproportionately affect underrepresented patient populations. Without globally representative training data, AI-based models risk exacerbating healthcare disparities rather than reducing them.

Data Privacy, Security, and Ethical Considerations: AI’s dependence on patient data, medical imaging, and electronic health records (EHRs) raises concerns about data security, privacy, and regulatory compliance. The integration of AI into clinical workflows must align with global data protection standards (e.g., GDPR, HIPAA) to ensure secure and ethical patient data management. Additionally, ethical concerns regarding AI-driven decision-making, liability in surgical outcomes, and transparency in AI recommendations must be addressed before widespread adoption.

High Implementation Costs and Resource Constraints: AI-powered technologies, particularly robotic-assisted surgery systems and AI-driven imaging tools, require significant financial investment, limiting their accessibility, especially in low-resource healthcare settings. The high costs of AI integration, infrastructure development, and continuous model updates present challenges in ensuring equitable AI adoption across different healthcare systems.

Learning Curve and Surgeon Adaptation: The integration of AI into surgical practice requires specialized training for surgeons, radiologists, and perioperative teams. The learning curve associated with AI-powered robotic surgery and decision-support tools may delay clinical adoption and necessitate structured training programs to ensure effective implementation. Additionally, AI-generated recommendations must be interpretable and actionable to gain trust among healthcare professionals.

Lack of Standardization in AI Regulatory Approval: There is no universal framework for AI validation, regulatory approval, or clinical deployment in thoracic surgery. The absence of standardized guidelines for AI-based surgical tools, decision-support systems, and diagnostic models leads to uncertainty in clinical adoption, requiring collaboration between regulatory bodies, researchers, and industry stakeholders to develop clear AI governance policies.

In addition to these limitations, it is important to note that this review included only studies published in the English language, which may have introduced a language bias and excluded relevant non-English literature. Furthermore, grey literature such as conference proceedings and non-peer-reviewed reports were not considered, potentially omitting emerging but unpublished insights. Another challenge lies in the rapid pace of technological advancement in the field of artificial intelligence, which may render some of the included studies quickly outdated as newer algorithms and systems are developed. Taken together, these limitations highlight the urgent need for more multicenter, prospective, and internationally representative studies using standardized AI evaluation frameworks. Such efforts will be critical for validating AI technologies across diverse clinical environments and ensuring their safe, equitable, and sustainable integration into thoracic surgical practice.

## 7. Conclusions

AI is rapidly transforming thoracic surgery, offering significant advancements in diagnostics, surgical precision, intraoperative guidance, and postoperative management. From AI-powered imaging analysis and robotic-assisted surgery to predictive analytics and real-time decision support, AI has the potential to enhance patient outcomes, optimize healthcare efficiency, and drive innovation in surgical practice. This review identified that AI applications in thoracic surgery are most advanced in diagnostic imaging, surgical planning, intraoperative guidance, and postoperative outcome prediction, with deep learning and machine learning models showing high accuracy and clinical utility across several domains. However, the studies reviewed were largely retrospective and heterogeneous, with limited prospective validation and regulatory readiness. These findings highlight both the growing capability and the existing gaps in translating AI into routine surgical practice. Addressing technical, ethical, and infrastructural challenges, as well as data security concerns, algorithmic biases, and the need for standardized clinical validation will be essential for enabling widespread, safe, and equitable adoption of AI in thoracic surgery. The successful integration of AI into thoracic surgery will require interdisciplinary collaboration, robust regulatory frameworks, and extensive multicenter validation to ensure its safety, efficacy, and reliability. To fully harness AI’s potential, future research must focus on improving AI interpretability, expanding dataset diversity, and enhancing AI-assisted training programs to support the next generation of thoracic surgeons. Ultimately, AI should be viewed as an augmentative tool—one that enhances rather than replaces the expertise of surgeons, supporting more precise, efficient, and patient-centered care. With continued technological advancements, ethical oversight, and clinical validation, AI is poised to revolutionize thoracic surgery, paving the way for safer, more effective, and highly individualized treatments.

## Figures and Tables

**Table 1:** Summary of biases identified in included studies.

Type of Bias	Description	Impact on Findings	Recommendations
**Selection Bias**	Overrepresentation of specific patient populations or disease stages due to non-randomized datasets.	Limits generalizability and external validity of AI model performance.	Promote inclusion of diverse, representative cohorts; use randomized or stratified sampling.
**Reporting Bias**	Incomplete or insufficient reporting of AI model development, training, and validation details.	Reduces transparency and reproducibility; impairs critical appraisal of findings.	Encourage adherence to AI-specific reporting guidelines (e.g., TRIPOD-AI, CONSORT-AI).
**Publication Bias**	Tendency to publish positive results, while negative or inconclusive findings are underreported.	Overestimates the perceived effectiveness of AI tools.	Register trials and encourage publication of all results regardless of outcome.
**Performance Bias**	Variability in the quality and size of training datasets used to develop AI models.	Increases risk of overfitting and poor generalization to external datasets.	Utilize larger, multicenter datasets; apply external validation methods.
**Detection Bias**	Inconsistent outcome measurement methods and validation techniques across studies.	Complicates study comparability; may distort conclusions about AI effectiveness.	Standardize outcome measures and validation protocols across studies.

**Table 2:** Overview of the studies included in this review on artificial intelligence applications in thoracic surgery. The table presents key details, including author(s), year of publication, study focus, AI application area, methodology, and main findings. The studies cover a range of AI applhighlights the growing impact of artificial intelligence (AI) in thoracic surgery, several limitations must ications, including early diagnosis, surgical precision, risk stratification, postoperative care, and oncological treatments.

Author(s)	Year	Title	Study Focus	AI Application	Key Findings
You et al. [18]	2022	Artificial intelligence in cancer target identification and drug discovery	Cancer target identification and drug discovery	Machine learning and network-based models for identifying anticancer targets and drug discovery	AI enables quantitative analysis of biological networks, identifying novel drug targets and promising drug candidates.
Zhang and Chen [19]	2022	Artificial intelligence: opportunities in lung cancer	Lung cancer management	AI in screening, diagnosis, and treatment, including clinical decision-support systems	AI shows significant potential across the lung cancer clinical pathway but requires advancements in interpretability.
Števík et al. [20]	2024	Hybrid AI solution combining CNN and analytical approaches in lung ultrasound for thoracic surgery	Lung ultrasound diagnosis	Convolutional neural networks (CNNs) combined with analytical approaches	Hybrid AI models demonstrated higher accuracy in detecting A-lines compared to radiology residents.
Woodhouse et al. [21]	2025	Leveraging AI as a safety net for incidental lung nodule identification	Lung nodule identification	AI-based safety systems for incidental findings in imaging	AI effectively reduces missed incidental nodules, enhancing detection rates.
Liang et al. [22]	2025	LungDiag: AI for respiratory disease diagnosis based on electronic health records	Diagnosis of respiratory diseases	AI analysis of electronic health records (EHRs)	AI models accurately diagnose respiratory diseases, demonstrating scalability across multiple centers.
Zumla et al. [23]	2024	The role of AI in the diagnosis, imaging, and treatment of thoracic empyema	Thoracic empyema	AI applications in imaging and treatment	AI offers significant diagnostic accuracy in imaging-based thoracic empyema management.
Yoshimura et al. [24]	2024	Diagnostic AI model predicts lymph node status in NSCLC	Lymph node status prediction in lung cancer	AI predictive models for lymph node involvement	AI demonstrated high accuracy in predicting lymph node status using simplified data inputs.
Ishiwata et al. [25]	2024	Deep learning-based prediction of nodal metastasis in lung cancer	Prediction of nodal metastasis	Deep learning applied to endobronchial ultrasound (EBUS) imaging	AI improved accuracy in detecting nodal metastasis compared to traditional methods.
Zhao et al. [26]	2025	Deep learning for gene mutation prediction in lung cancer	Gene mutation prediction	Deep learning applied to histological image analysis	AI achieved robust performance in predicting gene mutations associated with lung cancer.
Ismail et al. [27]	2025	AI-driven automated lung sizing from chest radiographs	Lung sizing pre-transplantation	Automated lung sizing from radiographs	AI significantly streamlined pre-transplantation workflows, reducing manual errors.
Sargiotis et al. [28]	2024	Predictive performance of AI models for post-transplant outcomes	Post-transplant outcomes	AI for prediction of heart and lung post-transplant health outcomes	AI accurately predicted post-transplant complications and recovery trajectories.
Tian et al. [29]	2023	ML-based prognostic models for post-lung transplantation	Prognostic modeling	Machine learning for long-term outcome prediction	AI-enabled models effectively identified patients at risk of adverse outcomes post-transplant.
Etienne et al. [11]	2020	Artificial intelligence in thoracic surgery: past, present, perspective, and limits	AI in thoracic surgery	Comprehensive review of AI applications	AI offers significant potential, but limitations in data integration and adoption persist.
Aleem et al. [30]	2024	Enhancing thoracic surgery with AI: A review of current practices and emerging trends	AI in thoracic surgery	Review of AI advancements and emerging trends	Identifies trends in AI applications and challenges for widespread integration.
Shigemura [31]	2020	Transforming diagnostics in lung transplantation with AI	Diagnostics for lung transplantation	AI applications for diagnostics, particularly in imaging	AI improves diagnostic accuracy in lung transplantation by incorporating real-time imaging analysis.
Mitzman et al. [32]	2023	Resident training in robotic thoracic surgery	Training in robotic thoracic surgery	AI for robotic surgery training	Highlights the role of AI in enhancing surgical training, particularly for complex robotic techniques.
Todd et al. [33]	2020	Risk Factors for Acute Rejection in the First Year after Lung Transplant	Lung transplant rejection	AI for identifying risk factors for acute rejection	Identified key risk factors for rejection using data from a multicenter cohort, improving predictive modeling.
Hu and He [34]	2021	Evaluation of the Postoperative Nursing Effect of Thoracic Surgery Assisted by AI Robot	Postoperative nursing care	AI robotic systems for monitoring and assistance	AI-assisted robotic systems improved postoperative nursing outcomes and reduced complications.
Li et al. [35]	2023	Day surgery unit robotics thoracic surgery: feasibility and management	Robotics in thoracic day surgery	Robotic systems for minimally invasive day surgery	Demonstrated the feasibility and benefits of robotic-assisted day surgeries, including reduced hospital stays.
Fairbairn et al. [36]	2023	Robotic Lobectomy	Robotic lobectomy	Robotic-assisted thoracic surgery	Highlighted the advantages of robotic lobectomy in terms of precision, safety, and recovery times.
Lazar and Hwalek [37]	2023	A Review of Robotic Thoracic Surgery Adoption and Future Innovations	Robotic thoracic surgery adoption	Overview of robotic surgery technologies and innovations	Reviewed the current adoption trends and potential future advancements in robotic thoracic surgery.
Seastedt et al. [38]	2023	Robotic Mediastinal Surgery	Robotic mediastinal surgery	Robotic platforms for mediastinal procedures	Discussed the effectiveness of robotic platforms for complex mediastinal surgeries, highlighting reduced complications.
Veronesi G [39]	2014	Robotic thoracic surgery: technical considerations and learning curve	Robotic thoracic surgery learning curve	Robotic-assisted surgery and surgeon training	Discussed the technical challenges and surgeon learning curves associated with robotic thoracic surgery.
Steenwyk and Lyerly [40]	2012	Advancements in robotic-assisted thoracic surgery	Robotic-assisted thoracic surgery	Innovations in robotic surgery	Provided a historical perspective on advancements in robotic-assisted thoracic surgery technologies.
Tane et al. [41]	2023	Console and bedside surgeon fused robot-assisted thoracic surgery	Robotic thoracic surgery	Fusion of console and bedside surgical roles in robotic thoracic procedures	Demonstrated improved workflow efficiency and precision by fusing roles of console and bedside surgeons.
Ostberg et al. [42]	2021	Machine learning: principles and applications for thoracic surgery	Machine learning applications in thoracic surgery	Principles and frameworks for machine learning in thoracic surgical settings	Highlighted practical applications and barriers to implementation of machine learning in thoracic surgery.
Herrera et al. [43]	2023	Development of a robot-assisted thoracic surgery (RATS) program	Program implementation for robotic thoracic surgery	Lessons learned from implementing RATS in 2500 cases	Provided practical insights into challenges and successes of large-scale RATS program adoption.
Wang and Wang [44]	2023	Current status and prospect of robot-assisted thoracic surgery: A bibliometric analysis	Bibliometric analysis of robotic thoracic surgery	Statistical analysis of robotic-assisted thoracic surgery trends	Identified trends, research gaps, and areas for future exploration in robotic-assisted thoracic surgery.
Cold et al. [45]	2024	Artificial Intelligence Improves Novices’ Bronchoscopy Performance	AI in surgical training	AI-assisted bronchoscopy simulation for novices	Demonstrated significant improvements in bronchoscopy performance and learning outcomes for novices.
Seastedt et al. [46]	2022	A scoping review of artificial intelligence applications in thoracic surgery	Scoping review of AI in thoracic surgery	Comprehensive review of AI applications across thoracic surgery	Identified key areas of AI utility, limitations, and future directions in thoracic surgery.
Esteva et al. [47]	2007	Neural networks and artificial intelligence in thoracic surgery	Early applications of AI in thoracic surgery	Neural networks for image analysis and decision support	Discussed foundational AI techniques and their potential for improving thoracic surgical outcomes.
Gencer and Aydin [48]	2023	Can ChatGPT pass the thoracic surgery exam?	AI for educational purposes	Assessment of ChatGPT’s ability to respond to thoracic surgery exam questions	Found ChatGPT to have limitations in specialized thoracic surgery knowledge but potential for educational support.
Jones et al. [49]	2020	Autonomously Driven: Artificial Intelligence in Cardiothoracic Surgery	AI in cardiothoracic surgery	Overview of autonomous AI systems in surgical decision-making	Explored the development and challenges of autonomous AI in cardiothoracic surgery.

**Table 3:** Summary of AI modalities and reported performance across thoracic surgery domains. The table summarizes key AI modalities, application areas, example studies, reported performance, and clinical relevance.

AI Modality	Application Area	Example Study	Reported Performance	Clinical Relevance
**Deep Learning (CNNs)**	Pulmonary nodule detection	Yang et al. [57]	Accuracy: 94.2%; AUC: 0.91	Improves early lung cancer detection and treatment planning
**NLP + Machine Learning**	Respiratory disease diagnosis (EHR)	Liang et al. [22]	F1 Score: 0.927 (Top 3 diagnoses)	Supports rapid and scalable diagnosis via EHR analysis
**Random Forest**	Post-transplant survival prediction	Tian et al. [29]	Stratified survival: 14.8 vs. 52.9 months	Assists in identifying high-risk transplant patients
**AI-Enhanced 3D Reconstruction**	Surgical planning and intraoperative guidance	Li et al. [107]	Dice score: 89.2%; time savings: 24 min	Optimizes anatomical accuracy and reduces operative time
**Deep Learning**	Gene mutation prediction (EGFR/KRAS)	Zhao et al. [26]	High accuracy for EGFR/KRAS prediction	Supports targeted therapy decisions in surgical oncology
**Hybrid AI (CNN + analytical)**	Lung ultrasound imaging	Števík et al. [20]	Sensitivity: 92.8%; Specificity: 83.4%	Improves diagnostic accuracy in intraoperative imaging
**Ensemble Deep Learning**	Immunotherapy response prediction	Saad et al. [93]	C-index for OS: 0.75; outperformed clinical risk factors	Enhances immunotherapy stratification for surgical candidates
**Machine Learning**	Preoperative risk assessment	Poullis [75]	Enhanced prediction vs. traditional models	Enables personalized surgical risk modeling
**AI-Based Radiomics**	Prognosis and staging via imaging	Zheng et al. [72]	AUROC: 0.83 (detection); 0.74 (nodal metastasis)	Improves staging accuracy and treatment selection
**Artificial Neural Networks**	Radiotherapy toxicity and outcome prediction	Chan et al. [103]	Accuracy: 64.7%; predictive for survival outcomes	Informs cardiac-sparing strategies during radiotherapy
**Computer Vision + ML**	Intraoperative skill assessment and guidance	Ostberg et al. [42]	Real-time feedback and skill tracking; qualitative validation	Improves surgical precision and supports team-based performance optimization
**AI-Powered Monitoring Systems**	Postoperative complication detection	Hu and He [34]	Reduced complication rates and enhanced vital sign stability	Enables early detection of postoperative issues and supports enhanced recovery protocols
**Predictive ML Models**	Postoperative survival and readmission risk	She et al. [79]	Outperformed TNM staging in survival prediction	Supports personalized follow-up strategies and resource allocation post-surgery
